# Implementation of a digital exercise programme in health services to prevent falls in older people

**DOI:** 10.1093/ageing/afae173

**Published:** 2024-08-08

**Authors:** Morag E Taylor, Meghan Ambrens, Helen Hawley-Hague, Christopher Todd, Jacqueline C T Close, Stephen R Lord, Lindy Clemson, Thomas Lung, David Berlowitz, Jannette Blennerhassett, Julia Dayhew, Ashley Gluchowski, Wendy Hodge, Pamela Johnson, Reena Lasrado, Marita Merlene, Lillian Miles, Sandra O’Rourke, Catherine M Said, Leanne White, Nicola Wilson, Avigdor Zask, Kim Delbaere

**Affiliations:** Neuroscience Research Australia, Falls, Balance and Injury Research Centre, Sydney, New South Wales, Australia; Population Health, Faculty of Medicine and Health, University of New South Wales, Sydney, New South Wales, Australia; School of Health Sciences, Faculty of Medicine and Health, University of New South Wales, Sydney, New South Wales, Australia; Neuroscience Research Australia, Falls, Balance and Injury Research Centre, Sydney, New South Wales, Australia; Population Health, Faculty of Medicine and Health, University of New South Wales, Sydney, New South Wales, Australia; School of Health Sciences, Faculty of Biology, Medicine and Health, The University of Manchester, Manchester, UK; National Institute for Health and Care Research, Applied Research Collaboration—Greater Manchester, The University of Manchester, Manchester, UK; Manchester Academic Health Science Centre, Manchester, UK; School of Health Sciences, Faculty of Biology, Medicine and Health, The University of Manchester, Manchester, UK; National Institute for Health and Care Research, Applied Research Collaboration—Greater Manchester, The University of Manchester, Manchester, UK; Manchester Academic Health Science Centre, Manchester, UK; Manchester University NHS Foundation Trust, Manchester, UK; Neuroscience Research Australia, Falls, Balance and Injury Research Centre, Sydney, New South Wales, Australia; School of Clinical Medicine, Faculty of Medicine and Health, University of New South Wales, Sydney, New South Wales, Australia; Neuroscience Research Australia, Falls, Balance and Injury Research Centre, Sydney, New South Wales, Australia; Population Health, Faculty of Medicine and Health, University of New South Wales, Sydney, New South Wales, Australia; Sydney School of Health Sciences, Faculty of Medicine and Health, The University of Sydney, Sydney, New South Wales, Australia; Centre of Excellence for Population Ageing Research, The University of Sydney, Sydney, New South Wales, Australia; Sydney School of Public Health, Faculty of Medicine and Health, The University of Sydney, Sydney, New South Wales, Australia; The George Institute for Global Health, University of New South Wales, Sydney, New South Wales, Australia; Austin Health, Heidelberg, Victoria, Australia; Physiotherapy, University of Melbourne, Parkville, Australia; Austin Health, Heidelberg, Victoria, Australia; Health Promotion, Northern NSW Local Health District, Lismore, New South Wales, Australia; National Institute for Health and Care Research, Applied Research Collaboration—Greater Manchester, The University of Manchester, Manchester, UK; School of Health & Society, University of Salford, Salford, UK; ARTD Consultants, Sydney, New South Wales, Australia; Mid North Coast Local Health District, New South Wales, Australia; School of Health Sciences, Faculty of Biology, Medicine and Health, The University of Manchester, Manchester, UK; National Institute for Health and Care Research, Applied Research Collaboration—Greater Manchester, The University of Manchester, Manchester, UK; Manchester Academic Health Science Centre, Manchester, UK; ARTD Consultants, Sydney, New South Wales, Australia; Neuroscience Research Australia, Falls, Balance and Injury Research Centre, Sydney, New South Wales, Australia; Neuroscience Research Australia, Falls, Balance and Injury Research Centre, Sydney, New South Wales, Australia; Physiotherapy, University of Melbourne, Parkville, Australia; Physiotherapy, Western Health, St Albans, Victoria, Australia; Australian Institute of Musculoskeletal Science, St Albans, Victoria, Australia; Neuroscience Research Australia, Falls, Balance and Injury Research Centre, Sydney, New South Wales, Australia; Health Promotion, Northern NSW Local Health District, Lismore, New South Wales, Australia; Research & Innovation Service, Mersey Care NHS Foundation Trust, Liverpool, UK; Health Promotion, Northern NSW Local Health District, Lismore, New South Wales, Australia; University Centre for Rural Health, School of Public Health, University of Sydney, Lismore New South Wales, Australia; Neuroscience Research Australia, Falls, Balance and Injury Research Centre, Sydney, New South Wales, Australia; Population Health, Faculty of Medicine and Health, University of New South Wales, Sydney, New South Wales, Australia

**Keywords:** implementation science, exercise, health behaviour, telemedicine, postural balance, adherence, aged, qualitative research, older people

## Abstract

**Background:**

*StandingTall* uses eHealth to deliver evidence-based balance and functional strength exercises. Clinical trials have demonstrated improved balance, reduced falls and fall-related injuries and high adherence. This study aimed to evaluate the implementation of *StandingTall* into health services in Australia and the UK.

**Methods:**

Two hundred and forty-six participants (Australia, *n =* 184; UK, *n =* 62) were recruited and encouraged to use *StandingTall* for 2 h/week for 6-months. A mixed-methods process evaluation assessed uptake and acceptability of *StandingTall*. Adherence, measured as % of prescribed dose completed, was the primary outcome.

**Results:**

The study, conducted October 2019 to September 2021 in Australia and November 2020 to April 2022 in the UK, was affected by COVID-19. Participants’ mean age was 73 ± 7 years, and 196 (81%) were female. Of 129 implementation partners (e.g. private practice clinicians, community exercise providers, community service agencies) approached, 34% (*n =* 44) agreed to be implementation partners. Of 41 implementation partners who referred participants, 15 (37%) referred ≥5. Participant uptake was 42% (198/469) with mean adherence over 6 months being 41 ± 39% of the prescribed dose (i.e. 39 ± 41 min/week) of exercise. At 6 months, 120 (76%) participants indicated they liked using *StandingTall*, 89 (56%) reported their balance improved (moderately to a great deal better) and 125 (80%) rated *StandingTall* as good to excellent. For ongoing sustainability, health service managers highlighted the need for additional resources.

**Conclusions:**

*StandingTall* faced challenges in uptake, adoption and sustainability due to COVID-19 and a lack of ongoing funding. Adherence levels were lower than the effectiveness trial, but were higher than other exercise studies. Acceptance was high, indicating promise for future implementation, provided sufficient resources and support are made available.

**Trial registration:**

Australian and New Zealand Clinical Trials Registry ACTRN12619001329156.

## Key Points

- *StandingTall*, an eHealth fall prevention exercise programme, improves balance and prevents falls and fall-injuries.
- Despite successes in controlled environments, translating research into real-world practice often presents challenges.
- In this study, *StandingTall* was acceptable, appropriate, feasible and safe but sustained implementation was not achieved.
- Adoption rates were lower than expected, with fidelity (adherence) highlighting the persistent gap between research and practice.
- Translating fall prevention research into practice for population benefit continues to be a challenge.

## Introduction

Falls and fall-related injuries impact both the health system and the older population along with their support networks. Falls are the second leading cause of unintentional injury-related deaths globally [1]. Worldwide, 37 million falls require medical attention each year, resulting in 36-million disability-adjusted-life-years lost annually [2]. These statistics underscore the need for effective, scalable and sustainable fall prevention strategies.

There is strong evidence that falls can be prevented using appropriately prescribed exercise programmes [3]. Exercise to prevent falls should include balance and functional strength exercises and be of sufficient dose (2–3 h per week, for at least 6 months) [3, 4]. Implementation and reach remain a challenge. Moreover, even when individuals do engage in exercise programmes, long-term adherence is often poor due to factors such as physical discomfort, competing commitments, psychological barriers and shifting priorities [5, 6]. These challenges highlight the need for approaches that are adaptable, personalised and sustainable with appropriate reach to achieve long-term fall and fall-injury reductions at the population level.

The *StandingTall* programme merges digital technology and behaviour change techniques to deliver tailored and progressive balance and functional strength exercise. *StandingTall* was specifically designed for older people and can be conducted in the person’s home without supervision. The programme, recommended at a dose of 2 h per week, reduced falls and fall-related injury over 2 years in a randomised controlled trial, with confirmed improvements in standing balance at 6 months [7]. Adherence rates in the *StandingTall* trial were good, with 80% of the participants completing an average of 105 min of exercise per week in the first 6 months, and 68% maintained an average of 114 min per week at 12 months despite minimal interaction with study staff [7]. This sustained engagement suggests potential integration of these exercises into participants’ lifestyles [7].

The objective of the current study was to evaluate the implementation of *StandingTall* into healthcare practices and services in Australia and the United Kingdom (UK). More specifically, we wanted to understand the factors influencing real-world uptake and adherence to *StandingTall*. During the trial, the COVID-19 pandemic forced a shift towards telehealth (eHealth) that allowed us to examine *StandingTall* in this context.

## Methods

### Study design

The study was designed as a multisite, pragmatic clinical implementation trial with process evaluation. A study protocol has previously been published [8].

### Sites

The trial included four sites: three in Australia and one in the UK. In Australia, two health districts in New South Wales [NSW; Mid-North Coast Local Health District (MNCLHD) and Northern NSW Local Health District (NNSWLHD)] and one site in Victoria (Austin Health) implemented *StandingTall*. In the UK, Northern England was considered one site with participants recruited from within the boundaries of Greater Manchester, North-West Coast and Yorkshire and Humber Clinical Research Networks [8].

### Participants

Participants were recruited through health services, community organisations and media advertisements. Inclusion criteria were community-dwelling, aged 60 years or older and sufficient English language skills to understand study documents. Exclusion criteria in brief (fully described in the protocol): residents of aged care facilities; medical conditions precluding safe exercise participation and mobility limitations (unable to walk 10 m indoors without the use of a walking aid) [8]. During the COVID-19 pandemic, telehealth set-ups were introduced that required additional inclusion criteria for safety [8].

### Recruitment

Study recruitment was conducted over different timeframes in Australia and the UK. In Australia, participants were recruited from October 2019 to March 2021, with all participants completing the study by September 2021. In the UK, participants were recruited from December 2020 to October 2021, with all participants completing the study by April 2022.

### Ethics and consent

Ethical approval was obtained from the South-Eastern-Sydney LHD Human Research Ethics Committee (HREC 18/288 approved 28/02/2019) in Australia and from North West-Greater Manchester-South Research Ethics Committee in the UK (IRAS: 268954 Approved 04/03/2020; with Health Research Authority approval 14/05/2020). Potential participants provided verbal consent to be screened for eligibility and written informed consent or informed online consent (during the COVID-19 pandemic), prior to study enrolment. Implementation partners (e.g. health professionals/exercise specialists, community referral agents) and support persons provided implied consent by clicking a link in an invitation email for online surveys and written informed consent or online informed consent (during the COVID-19 pandemic) for interviews.

### *StandingTall* intervention programme

A full description of the *StandingTall* programme is available in the protocol paper [8]. Figure S1 illustrates the user interface for the *StandingTall* programme and examples of the balance assessment and evidence-based balance and functional strength exercises. Before commencing the programme, participants completed online training and had to pass a quiz with 80% accuracy to gain access [8]. A set-up session with an exercise specialist experienced in delivering/prescribing exercise for older people was recommended [8]. Initially, this session was face-to-face, but transitioned to telehealth during the COVID-19 pandemic [8]. Participants were asked to use *StandingTall* for 6 months, starting at 40 min/week and increasing by 20 min each fortnight until reaching 120 min/week from Week 9 onwards. Exercise intensity was individually tailored [8]. Adherence data (exercise minutes per week) were automatically recorded and transferred to a secure server at Neuroscience Research Australia for remote access and monitoring. Participants were contacted by phone if their adherence dropped below 85% for two consecutive weeks, and a central study helpline was available for assistance [8].

Health professionals/exercise specialists, seen as implementation partners, played a key role by referring participants to the study, conducting set-ups and monitoring progress. They completed online training with an 80% pass rate to access the app’s content management system (‘back-end’). The back-end allowed for remote monitoring of exercise adherence and progression, as well as adjusting exercise categories and intensity (e.g. in the case of illness/holidays).

In Australia, part-time site-specific study staff (implementation officers) facilitated implementation through promotion, training and support of exercise specialists, in addition to handling participant screening, consent and adherence monitoring. In the UK, centrally employed study university-based staff promoted the programme and provided training and support to community exercise providers who were responsible for recruitment, screening, consent, set-ups and ongoing monitoring. These providers received a small fee for recruiting participants following standard UK Clinical Research Network (CRN) practice. The different approaches to study procedures reflect the distinct healthcare and research environments in Australia and the UK.

### Impact of bushfires, floods and COVID-19

The study faced significant challenges due to a series of natural disasters and the global COVID-19 pandemic, which affected the research process and participant/exercise specialist engagement at all sites. In brief, in NNSWLHD and MNCLHD from September 2019 to January 2020, there were bushfires and floods. At all sites from March 2020 to the completion of the study, the COVID-19 pandemic affected the study.

### Resources

A study website (www.StandingTall.org.au) assisted participants, support persons and health professionals/exercise specialists in using and delivering *StandingTall*. The website provided online training modules and resources (e.g. programme manual, fact sheets and quizzes) [8].

### Outcome measures

The nested process evaluation used quantitative and qualitative methods to explore uptake and acceptability of the *StandingTall* programme, providing guidance for scale-up [8].

The primary outcome was fidelity assessed by adherence to *StandingTall* during the 6-month study. Weekly exercise minutes (adherence) were monitored in the back-end and calculated by averaging the % of the weekly prescribed dose completed. Only actual exercise minutes were recorded excluding rest periods, watching instructional videos or set-up time, and before 16 February 2021, incomplete sessions were not recorded. For participants who withdrew from exercise or the study, exercise minutes were recorded as zero from the withdrawal point to avoid bias in adherence reporting.

Secondary process evaluation outcomes, based on the Proctor Framework [9], examined adoption, appropriateness, acceptability, feasibility, sustainability, implementation cost and adverse events using study logs and through surveys and/or interviews with participants, support persons, exercise specialists/healthcare workers and health service managers [8]. Participants were surveyed at baseline, 3 months and 6 months. Interviews were conducted via telehealth due to COVID-19. In Australia, ARTD (the independent external evaluator) conducted interviews with participants, implementation partners, managers, stakeholders and study staff. In the UK, study staff conducted interviews with participants and implementation partners via telehealth. Participants willing to be interviewed were sampled by age and sex by site. All willing implementation partners, managers and stakeholders with relevant experience were interviewed. These interviews also explored the barriers and facilitators to delivering the programme through telehealth in the context of the pandemic.

### Sample size calculation

Based on our primary outcome (adherence over 6 months), we aimed to recruit 100 participants per site (total *N =* 400) allowing for a 20% loss to follow-up (full details in protocol paper) [8].

### Statistical analysis

Quantitative and qualitative methods were used to explore study outcomes. Continuous outcomes, including adherence (% of prescribed dose completed; primary outcome) and exercise minutes, were reported as mean [standard deviation (SD)] and median [interquartile range (IQR)]. Survey responses and categorical data were reported as frequency and %. Missing data were not imputed (*n =* 15 for age, *n =* 3 for sex, *n =* 18 for fall history, *n =* 10 for joint problems and walking aid use, *n =* 14 for current employment status, *n =* 74 for 3-month survey and *n =* 88 for 6-month survey). A chi-square test compared self-reported balance change in groups with higher (≥22 h exercise over 6 months) and lower (<22 h exercise over 6 months) adherence. The 22 h, chosen a priori, equates to 60 min of exercise per week for 22 weeks, allowing 4-week holiday/illness during the 6-month trial. This was based on 6-month adherence data from the effectiveness RCT, considering total exercise time (including system navigation, watching instructional videos and rests) approximates to the recommended 2 h per week for fall prevention [7]. Interviews were audio-recorded, transcribed verbatim and thematically analysed in NVivo 14 until data saturation was achieved. A combination of inductive and deductive coding were used to organise data into major themes and subthemes related to barriers and facilitators at the participant, health professional/exercise specialist, health service manager and stakeholder and study staff levels.

## Results

Two hundred and forty-six older participants were recruited to undertake 6 months of exercise using *StandingTall*. Of these, 184 (75%) were from Australia (69 [38%] from NNSWLHD and 79 [43%] from MNCLHD, NSW and 36 [20%] from Austin Health, VIC) and 62 (25%) from Northern England, UK.

The participant flow through the study is illustrated in Figure S2. In Australia, 39 people were ineligible. In the UK, 9 people were ineligible with most participants recruited through community exercise providers’ existing clientele (Figure S2). In Australia, 184 of 403 (46%) eligible people participated in the study (Figure S2). In the UK, data on eligibility and participation were available for two of five sites, where 14 (58%) of 24 eligible people participated in the study (Figure S2). The reasons for nonparticipation are provided in Figure S2.

Participants were aged 73 years (SD 7), 196 (81%) were female, 46% (*n =* 112) reported a fall in the past year [34% (*n =* 17) in Northern England; 53% (*n =* 95) in Australia], 13% (*n =* 30) reported using a walking aid for daily life and 57% (*n =* 135) reported joint problems. Eighty-six percent (*n =* 199) were retired, 8% (*n =* 18) were employed and 7% (*n =* 15) volunteered in community activities/services. Fifty-four percent (*n =* 132) reported being born in Australia, 24% (*n =* 60) in the UK, 14% (*n =* 35) from various other countries and for 8% (*n =* 19), country of birth was not recorded.

### Primary outcome: fidelity (exercise adherence)

**Figure 1:**
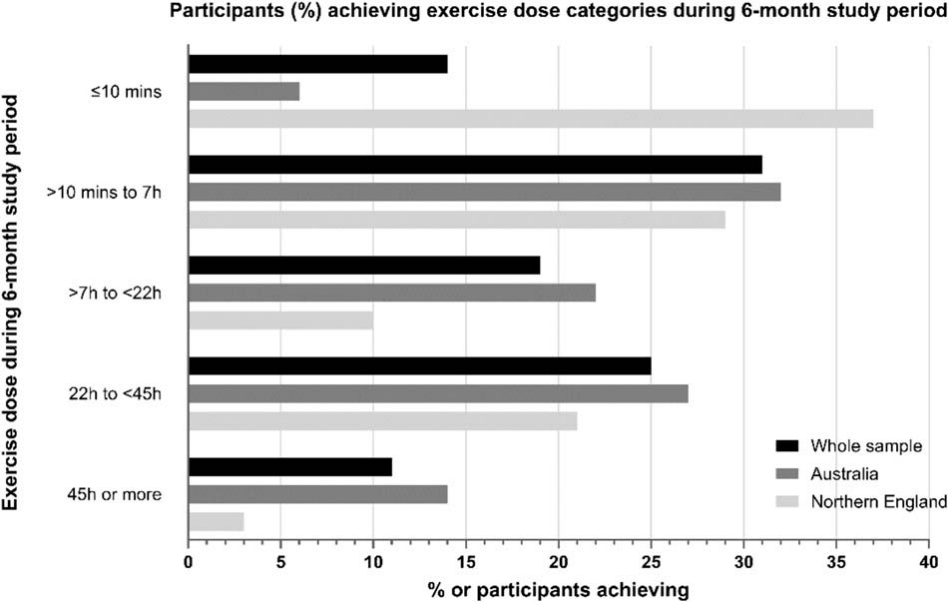
Proportion of participants undertaking various exercise doses during the 6-month study period.

Table 1 presents mean adherence, measured as the % of the prescribed dose completed, for Australian and UK sites, as well as adherence for the whole sample. In Australia, mean adherence over 6 months was 46% (SD 39) of the prescribed dose, with an average of 45 min/week (SD 42). In the UK, mean adherence was 24% (SD 33), with an average of 23 min/week (SD 35). Overall mean adherence was 41% (SD 39) of the prescribed dose, with an average of 39 min/week (SD 41). Mean and median adherence for Weeks 0–12 and 13–26 are reported in Table S1. Mean and median exercise minutes by week and for the 6-month study period are reported in Table S2. Adherence to exercise during the 6-month study period is illustrated in Figure 1.

**Table 1:** *StandingTall* implementation adherence for the 26-week study period.

Adherence^a^	26 weeks: whole sample			26 weeks: exercisers	
	Adherence, % Mean (SD)	Adherence, % Median [IQR]	People with no exercise minutes W1–26, *n*(%)^c^	Adherence, % mean (SD)	Adherence, % median [IQR]
NNSW (*n =* 69)	42 (40)	25 [6, 87]	2 (3)	43 (40)	26 [7, 88]
MNC (*n =* 79)	52 (37)	45 [19, 80]	3 (4)	54 (37)	47 [25, 84]
Austin (*n =* 36)	42 (39)	32 [5, 68]	3 (8)	46 (38)	36 [15, 77]
Australia (*n =* 184)	46 (39)	38 [9, 80]	8 (4)	49 (38)	40 [10, 85]
Northern England (*n =* 62)	24 (33)	5 [0, 48]	13 (21)	30 (35)	11 [1, 64]
Whole sample (*n =* 246)	41 (39)	31 [5, 74]	21 (9)	45 (38)	36 [7, 78]

^a^Exercise time is progressed in the first 9 weeks. Target dose: Week 1–2 = 40 min; week 3–4 = 60 min; Week 5–6 = 80 mins; Week 7–8 = 100 min and from Week 9 onwards = 120 min; adherence is reported as the % of the prescribed dose completed.

^b^Exercisers are defined as those participants who had exercise minutes recorded in the time frame reported.

^c^Initially, *StandingTall* did not record exercise minutes if the session was not completed; this was changed on 16 Feb 2021. In Australia, all of the people who recorded no exercise minutes from W1 to W26 started participating before this date and some (*n =* 3) have acknowledged poor adherence and therefore potentially did undertake some exercise, but the system did not record their session as it was incomplete. In Northern England, 5 of 13 people who recorded no exercise minutes from W1 to W26 started participating before this date and 1 acknowledged poor adherence and therefore potentially did undertake some exercise, but the system did not record their session as it was incomplete.

Of the participants who completed 22 h or more of exercise (*n =* 85), 69% (*n =* 59) reported at least moderately better balance at 6 months, compared to 41% (*n =* 30/73) of those who completed <22 h of exercise [chi^2^_(1, *n =* 158)_ = 12.8, *P* < .001].

Qualitative data suggest programme factors, including technological connectedness, and existing medical issues and busy schedules were barriers to adherence (Table S3). Health professionals/exercise specialists identified additional barriers: technology-related challenges (including technology skills and reduced technology confidence), patient medical issues as competing priorities, a preference for social interaction and the research-focussed study processes (Table S3).

### Secondary outcomes: implementation findings

Adoption, appropriateness, acceptability, coverage, feasibility, sustainability and adverse events are reported in Table 2. These secondary outcomes are also supported by qualitative evidence from interviews (Tables S4–S6). Tables S4–S6 provide illustrative quotes for each theme and subtheme developed by analysing 74 interviews with 35 participants (*n =* 20 Australian; *n =* 15 UK), 27 health professionals/exercise specialists (*n =* 19 Australian; *n =* 8 UK), five Australian health service managers, five study staff (*n =* 3 Australian, *n =* 2 UK) and two Australian stakeholders.

**Table 2:** Secondary implementation outcomes.

Generic implementation outcome	Related *StandingTall* outcome/s	Variables or measures^a^
Adoption	At-risk older people are being referred by many pathways	AUS: 33 implementation partners (services or organisations) across 3 sites and 3 community referral agents; 36 total UK: 5 implementation partners, i.e. Tier-2 early intervention services in Northern England Uptake by different implementation partners: see Table S4 for Australian data. UK participants were known to the community exercise providers without reliable ineligibility/nonparticipation information^a^
		Number of implementation partners referring to 5 or more older people enrolled in the study: *n =* 15 (37%) AUS *n =* 12 of 36 (33%) UK *n =* 3 of 5 (60%)
		Average hours per week provided by support persons to support participants’ use of *StandingTall*—not available^c^
		Average number of people supported by healthcare worker in 18-month period—not available^c^
		Uptake of participants: 42% (198/469; includes ineligible for AUS; data for 2/5 UK sites only and does not include ineligible participant who was not reliably recorded: 14 of 24 participated) or 46% (198/427) of eligible participants. AUS: 184 of 445 older people referred were accepted into study [41% uptake or 46% (184/403) of those eligible to participate] UK: this cannot be accurately calculated, as reasons for nonparticipation were only available for 2 sites: 14 (58%) of 24, and ineligibility was not reliably recorded^b^
Appropriateness	Education modules meet stakeholders’ information needs Support persons are actively engaged in *StandingTall*, understand role	95% (*n =* 20) exercise specialists completing website satisfaction survey rate instruction modules on the *StandingTall* website as excellent or good quality 100% (*n =* 24) *StandingTall* users completing website satisfaction survey rate instruction modules on the *StandingTall* website as excellent or good quality
		3-month survey: 91% (*n =* 157) participants agree/tend to agree ‘I find *StandingTall* is easy to use’ (AUS/UK combined)^b^ 6-month survey: 92% (*n =* 145) participants agree/tend to agree ‘I find *StandingTall* is easy to use’ (AUS/UK combined)^a^
		% support persons rate *StandingTall* as good or somewhat good fit with normal practise—not available^c^
		100% (4 of 4) support persons agree the instruction modules on the *StandingTall* website are good quality.
Acceptability	At risk older people understand the benefits, how to use the programme and about supports	3-month survey: 84% (*n =* 144) participants agree/tend to agree ‘I like to use the *StandingTall* programme’ (AUS/UK combined)^b^ 6-month survey: 76% (*n =* 120) participants agree/tend to agree ‘I like to use the *StandingTall* programme’ (AUS/UK combined)^a^
		3-month survey: 80% (*n =* 137) participants rate *StandingTall* as good or excellent (AUS/UK combined)^b^ 6-month survey: 80% (*n =* 125) participants rate *StandingTall* as good or excellent (AUS/UK combined)^a^
		% hospital and community workers involved in study that rate *StandingTall* as a good or excellent falls prevention intervention—not available^c^
		% support persons agree that *StandingTall* has had a positive or somewhat positive impact on their professional practice—not available^c^
Coverage	At-risk older people engage with *StandingTall*, respond to feedback	Number people enrolled in *StandingTall* by age and gender, at each study site and in total: 81% (*n =* 196) of participants were female, and the average age was 73 years (SD 7). The youngest participant was 60 years and the oldest 93 years. AUS: age 73 ± 7 years, *n =* 148 (81%) UK: age 73 ± 7 years, *n =* 48 (80%)
Feasibility	Partner organisations/services commit to study Support persons give participants sufficient support At risk older people engage with *StandingTall*, respond to feedback	% hospital and community-based workers who regularly monitor patient’s progress on *StandingTall* exercise programme—not available^c^
		In lieu of surveys, from interviews: limited use of the content management system designed to monitor *StandingTall* users’ progress across 2 of 3 Australian sites. MNC LHD where *StandingTall* agreements were in place with physio services; 33% (2 of 6) clinicians interviewed regularly monitored patient progress using the content management system
		AUS: 36 (32%) services/partners agree to be involved from 111 approached potential partners included exercise specialists (physios/EPs) working in hospital departments, out-patient clinics/programmes and private settings; libraries; aged care providers; community groups for older people. There was variable involvement depending on the site. UK: 8 (44%) services agreed to be involved from the 18 approached; however, only 5 (28%) actually participated in the study (COVID-19). Potential partners included falls teams, community rehabilitation teams and exercise specialist teams (leisure services, AgeUK—charitable organisations or private services who were commissioned by the health service). There was variable engagement across the sites that did engage.
		3-month survey: 94% (*n =* 162) participants agree/tend to agree ‘I feel confident about doing the *StandingTall* exercises’ (AUS/UK combined)^b^ 6-month survey: 94% (*n =* 149) participants agree/tend to agree ‘I feel confident about doing the *StandingTall* exercises’ (AUS/UK combined)^a^
Sustainability	*StandingTall* referral pathways integrated into business as usual	% hospital and community-based workers who state they are definitely or probably going to keep using *StandingTall* as part of their patient care—not available^c^
		In lieu of surveys, from interviews: 25 of 27 (93%) health professionals/exercise specialists indicated they would continue to use *StandingTall* if it were rolled out (AUS/UK combined)
		Number of health service managers’ who state that the programme is being supported by relevant clinicians and partner organisations for continued use of *StandingTall* programme^c^
		AUS: 4 out of 5 (80%) of health service managers anticipate that *StandingTall* could become part of business as usual but only if dedicated resources for set-up and support are made available UK: health service managers conceived *StandingTall* as being most useful as a tool to prescribe home-based exercise for those who have been discharged from their service or unable to participate
Implementation cost		Australian health service managers saw a need for some level of funding to support a position to coordinate *StandingTall* delivery
		6-month survey: 46% (*n =* 72) participants were willing to pay a small one-off amount of $5/£3 to access the *StandingTall* programme (AUS/UK combined)^b^ 6-month survey: 23% (*n =* 37) may be willing to pay (AUS/UK combined)^a^
		6-month survey: 17% (*n =* 26) participants interested in accessing the programme and advice from a helpline service as part of an annual subscription (AUS/UK combined)^b^ 6-month survey: 36% (*n =* 56) may be willing to have an annual subscription (AUS/UK combined)^a^
Adverse Events^a^		Adverse events while exercising using the *StandingTall* programme or thought to be directly related to the *StandingTall* programme. AUS: *n =* 5; UK: *n =* 4. Adverse events were generally an exacerbation of joint pain that did not have lasting impact. There were 2 near falls while using the programme.

Note. AUS, Australia; UK, Northern England, United Kingdom

^a^Uptake data for Northern England is incomplete; only 2 of 5 sites provided uptake data. Most participants were approached directly by their clinician/provider, and therefore, referrals were mostly made to eligible people without reliable data collected for ineligibility. Very few potential participants self-referred that were unknown to the service.

^b^Participant survey completion *n =* 172 at 3 months and *n =* 158 at 6 months.

^c^A planned survey of participating healthcare workers was not completed because many who signed up initially to become implementation partners had minimal involvement after COVID-19 disrupted normal health service delivery. The issues covered in the survey were discussed in interviews with health workers who had sufficient involvement in *StandingTall* to comment in detail.

#### Adoption

Potential implementation partners included falls teams, community rehabilitation and exercise specialist teams [e.g. leisure services, AgeUK, exercise specialists (physiotherapists/exercise physiologists/fitness leaders) in hospital departments, out-patient clinics or private settings], libraries, aged care providers and community groups for older people. Of the 129 organisations approached across all sites, 44 (34%) agreed to be implementation partners with 41 providing referral pathways for their patients to use *StandingTall* and participate in the study. Most implementation partners were from Australian sites (*n =* 36; 32% adoption). In the UK, 8 (44%) of 18 organisations approached agreed to be implementation partners but only 5 (28%) referred participants due to changes in service provision during the COVID-19 pandemic. Fifteen of 41 (37%) implementation partners referred five or more participants. One site in the UK contributed 73% (*n =* 45) of the UK study participants, whereas in Australia, participant recruitment was more evenly distributed across the three sites (20%–43%).

Uptake was 41% (or 46% of eligible participants) in Australia and, with limited data available, 58% (14 of 24 with data) in Northern England (Table 2). For Australia, the referral pathways and uptake by referral pathway are reported in Table S4. The highest proportion of referrals and uptake came from community and self-referrals (31% of 445 referrals with 40% uptake). In the UK, all but six referrals came from community exercise providers who are referred patients from general practitioners or National Health Service (NHS) community falls and rehabilitation teams. The remaining six self-referrals were out of area for the study or in an area without a *StandingTall* community exercise provider.

Interviews with participants, health professionals/exercise specialists, health service managers and study staff identified various barriers and facilitators to the adoption of *StandingTall*. Representative quotes highlighting these barriers are detailed in Table S5. Barriers included: personal challenges (e.g. health, time); equipment needs (e.g. device, step and foam), resource constraints and internet access and stability; technical literacy and inequity related to eHealth; the delay in evidence for efficacy (trial started October 2019; efficacy RCT published April 2021 [7]); the constraints associated with research; and the lack of resources and support at all levels of healthcare, including government. Facilitators included: being evidence-based; user friendly design; low cost; improved reach; access to patient progress data; complements existing clinical care; progression from supervised programmes; helps meet quality accreditation; internal motivators such as concern about falls; and improved patient outcomes. *StandingTall* met an unmet need by serving as an alternative to traditional programmes (e.g. supervised group-based and home paper-based programmes), enabling participants to exercise at home through a tailored and progressive programme without supervision.

#### Appropriateness

Most exercise specialists rated the instruction modules on the *StandingTall* website as excellent or good quality (Table 2). Most participants agreed/tended to agree that *StandingTall* was easy to use (Table 2).

#### Acceptability

*StandingTall* was well received by participants, with 80% rating it as good or excellent at 3 and 6 months. More than 75% agreed or tended to agree that they liked using the programme at 6 months (Table 2).

Interviews with participants, health professionals/exercise specialists, health service managers and study staff identified some barriers and facilitators affecting acceptability. Representative quotes highlighting these barriers are detailed in Tables S6 and S7. Barriers included perceptions that *StandingTall* might not suit everyone (e.g. the lack of social interactions and upper limb strength exercises, very active people). Some found the programme somewhat rigid and repetitive, requiring additional support to maintain long-term engagement. The set-up process was considered too time-consuming, especially with the shift to telehealth during the pandemic, which disrupted the usual clinical schedule. The monitoring model did not align with exercise specialists’ usual care model, including aspects such as IT support and remote and longer-term monitoring, which some health professionals/exercise specialists felt were outside their usual scope of practice. Additionally, exercise specialists wanted more flexibility, such as the ability to disable specific exercises rather than categories. Facilitators included its ease-of-use, clear instructions and improvements in health and confidence. Participants appreciated the flexibility of completing exercises at their convenience, the tailored progression, the comprehensive exercise library—including cognitive-motor (brain training) exercises—and the evidence base behind the programme. The compatibility with telehealth was also seen as a facilitator, extending reach.

#### Coverage

The demographic and participant characteristics represent a cross section of the older population living in the community in both urban/metropolitan (e.g. Melbourne and Manchester) and regional areas (e.g. Lismore, Northern Rivers) (Table 2).

#### Feasibility

Thirty-four percent of approached implementation partners agreed to provide a referral pathway for the *StandingTall* programme (Table 2). Participant confidence in the programme was high, with most participants agreeing or tending to agree that they feel confident doing the *StandingTall* exercises (Table 2).

#### Sustainability

In Australia, most health service managers agreed that *StandingTall* could become embedded into usual care, though they highlighted the need for additional resources for set-up, monitoring and ongoing support (Table 2). UK managers perceived *StandingTall* as particularly beneficial postdischarge or for people unable to attend in-person sessions, but this may have been influenced by the COVID-19 pandemic.

#### Safety

Only minor adverse events were reported (Table 2).

#### Telehealth

The implementation of *StandingTall* through telehealth became more acceptable during the pandemic as a viable delivery method for health services (Table S7). However, adapting to a remote set-up presented challenges, especially when it diverged from the health services’ traditional service models (e.g. Wi-Fi/internet access and stability; Table S6 and S7). Technology access and technical literacy were also a concern (Table S7). Many health professionals/exercise specialists viewed any change to current practice as a barrier (Table S7). Some health professionals/exercise specialists commented on the importance of the social aspect of exercise in face-to-face sessions for long-term adherence (Table S3). However, they also acknowledged the long-term benefits, particularly in terms of accessibility, reach (to geographically dispersed areas) and maintenance (Table S7).

### Secondary outcomes: survey results

The complete 3-month (*n =* 172) and 6-month (*n =* 158) survey responses are reported in Table S8. At 3 months, 43% of participants reported their overall balance was ‘moderately to a great deal’ better, increasing to 56% at 6 months. At 3 and 6 months, most (80%) participants rated *StandingTall* as good or excellent overall. Most participants tended to agree or agreed that *StandingTall* was easy to use (91% and 92% at 3 and 6 months), that they liked to use the programme (84% and 76% at 3 and 6 months) and felt confident about doing the exercises (94% at 3 and 6 months). Ninety-two percent tended to agree or agreed that the instructions were helpful at 3 months and 74% and 76% thought the instructions were ‘about right’ at 3 and 6 months, respectively. The iPad was the most common device used for accessing *StandingTall* with most participants reporting no difficulty using devices to access the programme (74% and 79% at 3 and 6 months). The most common (48% and 50% at 3 and 6 months) difficulty was ‘doing 2 hours of exercise a week using *StandingTall’.* Just under half were willing to pay a small one-off fee to access *StandingTall* and approximately one-quarter potentially willing. An annual subscription was less popular (9% and 17% interested at 3 and 6 months; 47% and 36% potentially interested at 3 and 6 months).

## Discussion

This implementation study demonstrated that *StandingTall,* a cost-effective fall prevention exercise programme delivered using technology [7, 10], is appropriate, acceptable, feasible and safe. However, uptake was 46% and adherence levels were lower than expected and variable amongst participants. While many healthcare workers indicated they would continue using *StandingTall* if it were available, the programme was not embedded into usual care or the health system affecting sustainability. Despite health professionals/exercise specialists recognising the programme’s potential to complement current clinical care and address an unmet need in fall prevention, adoption of the programme was lower than anticipated. Approximately half of the participants were willing to pay a small one-off fee to access *StandingTall*, with many currently accessing or able to access free exercise programmes through their health service.

The study was substantially impacted by the COVID-19 pandemic. In the UK, the study start was delayed and three sites were unable to recruit. In both Melbourne and the UK, staff were redeployed or furloughed. In Australia, the study was suspended for a period of 3–4 months, which negatively impacted health worker engagement and participant recruitment and uptake.

The pandemic also forced a transition from face-to-face to telehealth set-ups requiring additional training, placing additional demands on already pressured healthcare workers and requiring additional resources (e.g. Wi-Fi). At times, this shift was incongruent with clinical practice procedures, where in-person sessions were preferred, as was the case in regional NSW where face-to-face sessions resumed but virtual set-ups for research had to continue. These challenges likely contributed to the lower participant uptake in the current study, with 21% of potential participants who provided a reason for non-participation choosing alternate exercise or citing a preference for face-to-face, tech apprehension, privacy concerns and study procedures as reasons for not participating. This aligns with a recent study showing that 41% of patients perceived telehealth as lower quality than traditional methods and 53% were unlikely to choose telehealth postpandemic [11]. Of note, in the Bennell et al. study, the participants were younger and only one-third were ≥60 years of age. Similar sentiments have been expressed by physiotherapists [12]. On the other hand, the pivot to telehealth/virtual set-up created new opportunities. Health service managers were particularly enthusiastic about using technology to improve health outcomes. For example, participants who may have ordinarily opted for group exercise were willing to try *StandingTall* during times of mandated home quarantine. Participants appreciated being able to access the programme from home, finding it a purposeful activity during lockdowns. Additionally, at two Australian sites, funds usually allocated to a face-to-face group-based fall prevention programme were redirected to support the implementation of *StandingTall*. For health service managers, telehealth provided flexibility and extended the reach of fall prevention to geographically dispersed regions. Highlighting the benefits of telehealth, while addressing any barriers related to technology and ensuring alignment with clinical practice, could further enhance participation.

While the target dose for *StandingTall* was 2 h/week from week 9 onwards (45 h total for 6 months), the recorded exercise minutes excluded the time spent navigating the programme, viewing instructional videos and rests. As a result, participants might have spent 25–30 min to accrue 15 min of actual exercise counted towards their total exercise time. Comparatively, adherence is commonly recorded as sessions attended that can inflate adherence estimates compared to *StandingTall’s* precise measurement [13, 14]. Additionally, before 16 Feb 2021, *StandingTall* only recorded exercise minutes from completed sessions. This meant incomplete sessions, like those paused for longer than 30 min or interrupted by internet issues for web users, were not recorded. This affected 181 (98%) Australian and 24 (39%) UK participants who started before this date, potentially impacting adherence rates. Unfortunately, we cannot quantify the impact of this change.

Adherence to the programme varied both among participants and between Australia and the UK, likely reflecting differences in implementation approaches. In Australia, local implementation officers acted as *StandingTall* champions, providing support to exercise specialists and participants and contacting participants if their adherence fell below 85% for two consecutive weeks. In contrast, in the UK, central study staff trained community exercise providers who then undertook all study activities, including recruitment (for which they received a small payment) and follow-up (which was variable). These operational differences likely explain the differences in adherence between Australia and the UK. The Australian more hands-on, supportive approach likely fostered better adherence by ensuring participants felt assisted and encouraged. This underscores the importance of a local champion to provide support, alongside the strategic use of behaviour change techniques to encourage continued participation [14, 15]. Participants’ fall history, which was higher in Australia, may have impacted their motivation to adhere, particularly as some participants felt the programme improved their confidence and awareness.

Overall, 11% of participants were fully adherent, which is less than the effectiveness trial (40% at 6 months) and a previous meta-analysis (21% at 6 months) [7]. However, about one-third of participants completed a therapeutic dose of 22 h of exercise over 6 months, surpassing adherence rates seen in the meta-analysis. This allowance accounts for the total exercise session time including transitions, rests and time watching instructional videos, likely amounting to 1.5–2 h. A greater proportion of participants who completed 22 h or more reported moderate to considerable improvements in balance at 6 months compared to those who completed less than 22 h, supporting its potential effectiveness at this dose.

The study identified areas for improvement in both the programme and set-up procedures, leading to several proposed enhancements. Some improvements were implemented during the trial (e.g. recording of exercise minutes for incomplete sessions), while others have been addressed post-trial or are ongoing. In June 2020, the programme introduced a broader range of starting intensities*,* expanding from two to five levels, facilitating a more engaging and appropriate challenge for participants with better initial balance. Feedback from exercise specialists prompted improvements in set-up efficiency and enhanced control over exercise tailoring post-trial. While exercise specialists could control some aspects of exercise prescription (e.g. override intensity settings, remove block/foam exercises), other aspects like removing specific exercises requires significant redevelopment. Participants also suggested that the selected exercise time (e.g. 10/15/20 min) should more accurately reflect the total time spent on the programme, including navigation, watching instructional videos and rests, to align expectations with actual exercise time.

*StandingTall* was not embedded into usual care at any site participating in the implementation study. The engagement of referrers was lower than expected, with only one-third referring five or more participants. Interviews with exercise specialists indicated a willingness to continue using *StandingTall* if available, but site managers suggested that additional funding would be needed for set-up and ongoing support. More work is needed to understand the best implementation model for *StandingTall,* particularly as only half of the participants were willing to pay a small fee to use the programme and a further quarter were willing to consider this. Potential strategies include a user-directed approach (i.e. simplifying the set-up process to remove the need for an exercise specialist) that would enable direct access for older people. Alternatively, a centralised set-up and support service could streamline the process for both users and healthcare providers, contingent on funding. Finally, commercialisation or continued pursuit of government support for health service and health promotion implementation could facilitate its long-term viability.

The remote monitoring features of *StandingTall* were not used to their full potential as traditional clinical practice and funding models, typically prioritising face-to-face sessions for exercise review and progress tracking. For remote monitoring to be viable, several factors need to be addressed [16]. In private practice, exercise specialists need to be appropriately reimbursed for remote consultations, including telephone follow-up [11, 16, 17]. Public hospital settings, which usually limit the number of sessions before discharging patients, might need to adapt to accommodate longer-term monitoring. Additional training and familiarisation with the programme’s back-end for exercise specialists could facilitate remote monitoring, alongside improvements in its user-friendliness, ease of navigation, automation (e.g. an opt-in approach for email progress updates to exercise specialists) and accessibility (e.g. streamlined access to crucial information) [16]. Additionally, some users may prefer not to be monitored and health literacy could be promoted to encourage self-monitoring [18].

The study faced some limitations. Firstly, the study was significantly impacted by the COVID-19 pandemic and two natural disasters in the regional NSW sites during recruitment. Secondly, the reliance on participant-completed surveys for data collection could have introduced a positive bias, with participants who had a favourable experience being more likely to complete the surveys. Thirdly, the results of the randomised controlled trial providing evidence for effectiveness were not published until 6 April 2021, which was after the last participant was recruited in Australia and halfway through the study period in the UK, and this may have impacted exercise specialist adoption and uptake [7]. Finally, although only 9% (4% Australia; 21% UK) of participants recorded no exercise minutes for the entire 6-month period, a significant proportion stopped exercising by Week 13. In Australia, just over one-third had no exercise minutes for Weeks 13–26, while in the UK, this was twice as high with two-thirds recording no exercise minutes for the same period. This likely resulted from different implementation approaches and underscores the need for ongoing support and monitoring to promote sustained engagement and adherence in unsupervised eHealth exercise programs for older people.

For the successful clinical rollout of the programme, access to suitable technology and reliable internet is essential, which can vary by demographic characteristics, socioeconomic status and geographical area [19]. People also need to have the motivation, capacity and confidence to use technology or have support available to navigate digital barriers—a known problem for older people [20]. Preferably, users should have an engaged exercise specialist for ongoing monitoring, feedback and encouragement to sustain programme engagement. To better fit with clinical practice time constraints, a shorter set-up session has been designed. Future improvements to increase programme uptake and acceptability might include tailoring exercises more closely (e.g. selecting or removing individual exercises), ensuring session time accurately reflects actual exercise time and automated and enhanced monitoring features for a more user-friendly experience. Adding a community component for peer support and a centralised support system may help users feel more supported. Finally, government policies supporting fall prevention and the removal of certain research-related study procedures (e.g. eligibility criteria, consent, surveys) could improve integration into routine care and uptake.

In conclusion, *StandingTall* was acceptable, appropriate, feasible and safe for use. Feedback from users and health professionals/exercise specialists has led to numerous improvements, with some already implemented and others in progress. Nonetheless, further work is needed to improve engagement, adherence, adoption and sustainability. Determining the best implementation strategy for *StandingTall* as a cost-effective fall prevention intervention is crucial. Programme improvements are expected to increase adherence and adoption. However, for *StandingTall* to be sustainable and widely implemented, supportive strategies such as commercialisation and/or government funding and support need to be further explored and developed. With the right adjustments and support, *StandingTall* has the potential to become a cost-effective fall prevention intervention at the population level, targeting older people and those at risk of falls.

## Supplementary Material

aa-24-0740-File002_final_afae173
