# Supplementary material for: Implementation of a digital exercise programme in health services to prevent falls in older people
Source: Age Ageing. 2024 Aug 8;53(8):afae173. doi: 10.1093/ageing/afae173 (PMC11306314; doi:10.1093/ageing/afae173)
Supplement: aa-24-0740-File002_final_afae173 [file aa-24-0740-file002_final_afae173.docx]

**Supplementary material: Implementation of a digital exercise programme in health services to prevent falls in older people**

**Table of contents**

[Supplementary Figure 1. An illustration of the user interface for the StandingTall programme 2](#_Toc173749654)

[Supplementary Figure 2. Study flow 3](#_Toc173749655)

[Supplementary Table 1. *StandingTall* Implementation Adherence for the 1-12 week and 13-26 week study periods 4](#_Toc173749656)

[Supplementary Table 2. Exercise minutes per week by site, country and total 5](#_Toc173749657)

[Supplementary Table 3. Exploring adherence to the *StandingTall* programme 6](#_Toc173749658)

[Supplementary Table 4. Referral source and uptake for Australian sites 8](#_Toc173749659)

[Supplementary Table 5. Exploring the uptake and adoption of the *StandingTall* programme to provide direct guidance for programme scale up 9](#_Toc173749660)

[Supplementary Table 6. Exploring the acceptability (i.e. do they like it) of the *StandingTall* programme to provide direct guidance for programme scale up 16](#_Toc173749661)

[Supplementary Table 7. What were the barriers and facilitators to telehealth delivery (because of COVID-19) 20](#_Toc173749662)

[Supplementary Table 8. Survey outcomes for 3- and 6-months 24](#_Toc173749663)

Supplementary Figure 1. An illustration of the user interface for the StandingTall programme: A. Selecting a balance assessment, the safety checklist and two examples of balance assessments – feet together on floor and near tandem on foam;

B. Selecting an exercise session, an example of a balance exercise, a how-to video and a how-to quick refresh; and


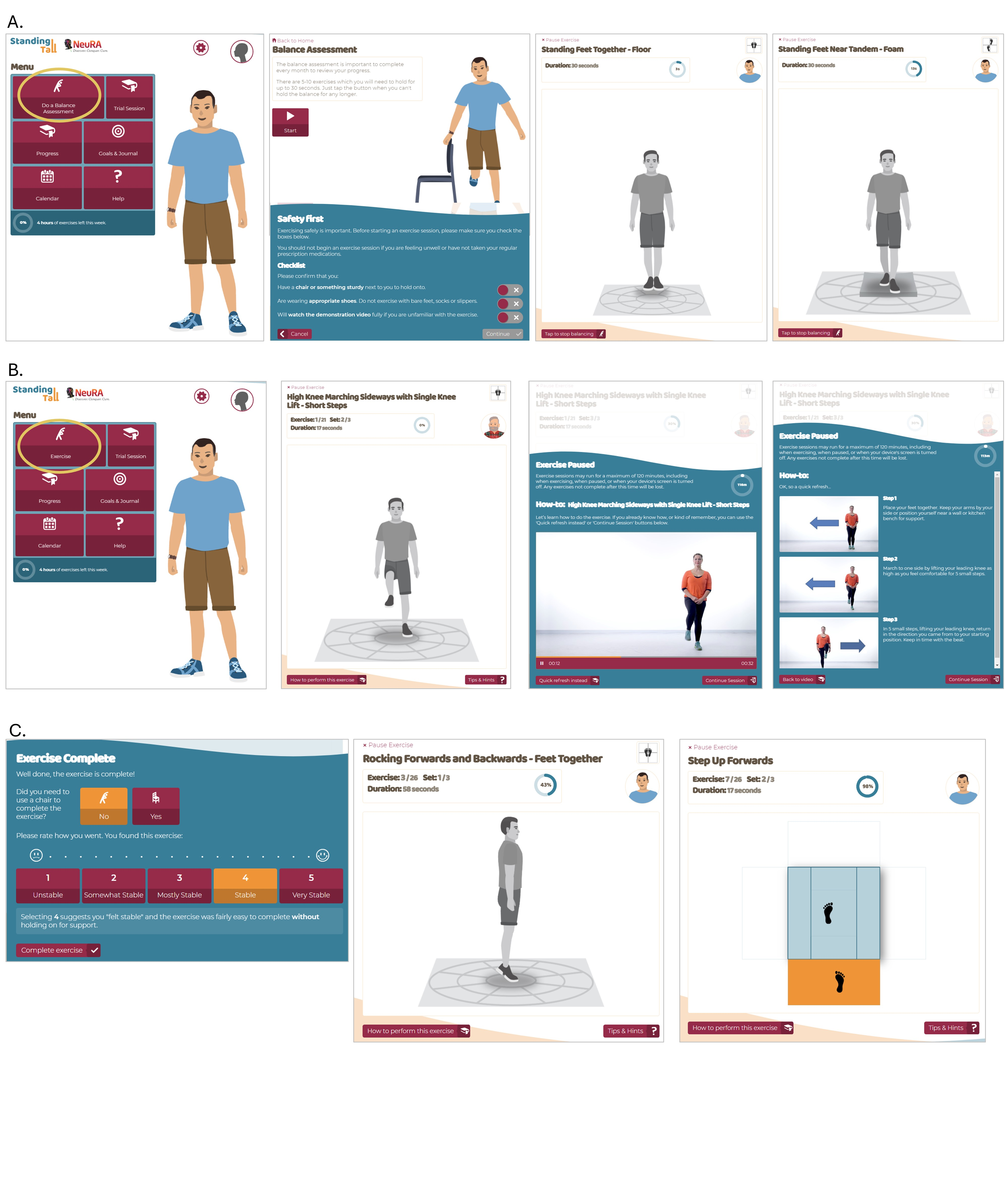
C. The exercise intensity rating scale and two examples of functional strength exercises

## Supplementary Figure 2. Study flow

**Enrolled in study**

Total: n=246

Australia: n=184

UK: n=62

**Baseline survey**

Total: n=225

Australia: n=178

Northern England: n=47 (45 exercise)

**3-month survey**

Total: n=172

Australia: n=149 (20 WD; 15 MD)

UK: n=23

**Ineligible:** n=42 AUS; n=9 UK^a^

Too young: n=2 AUS; n=1 UK

Neurological condition: n=17 AUS; n=2 UK

Not community-dwelling: n=1 AUS

No device/WiFi: n=14 AUS

Unable to walk 10m indoors: n=3 AUS

Vision impairment: n=2 AUS

Psychological condition: n=1 AUS

Not living within study area: n=2 AUS; n=6 UK

**Reasons for non-participation:** n=219 AUS; UK = 10^b^

Not interested: n=22 AUS

Not necessary: n=6 AUS

Too busy/can’t commit: n=17 AUS

Unwell/health issues: n=33 AUS

Already exercising: n=13 AUS

Chose alternate exercise: n=3 AUS; n=10 UK

Prefers F2F: n=8 AUS

Unforeseen circumstances^c^: n=8 AUS

Tech apprehension/issues: n=10 AUS

Travelling: n=8 AUS

Privacy concern: n=1 AUS

Study procedures: n=4 AUS

COVID-19 suspension/delay: n=14 AUS

Did not say or could not contact: n=71 AUS

**Potential participants**

Total: n=526^a,b^

Australia: n=445

UK: n=81^a,b^

**6-month survey**

Total: n=158

Australia: n=142 (26 WD; 16 MD)

UK: n=16

**Withdrew from study**

Total: n=42 (17%)

Australia: n=26 (14%)

UK: n=17 (27%)

**Withdrew from exercise only**

Total: n=48 (20%)

Australia: n=24 (13%)

UK: n=24 (39%)

Notes. AUS = Australia; F2F = face to face; Tech = technology; UK = United Kingdom

^a^In Northern England, UK, clinicians predominantly approached eligible participants with very few community/self-referrals and ineligibility was not routinely recorded.

^b^Reasons for non-participation only available for 2 of 5 sites (n=14/24 participated) in the UK

^c^Unforeseen circumstances e.g. death in the family, moving house, caring for family

## **Supplementary Table 1**. *StandingTall* Implementation Adherence for the 1-12 week and 13-26 week study periods

| Adherence^a^ | 12-weeks: whole sample^b^ | | |  | 12-weeks: exercisers^c^ | |  | 13-26-weeks: whole sample^b^ | | | |  | | 13-26-weeks: exercisers^c^ | | |
| --- | --- | --- | --- | --- | --- | --- | --- | --- | --- | --- | --- | --- | --- | --- | --- | --- |
|  | Adherence, %  Mean (SD) | Adherence, %  Median [IQR] | People with no exercise minutes W1-12, n(%)^c^ |  | Adherence, %  Mean (SD) | Adherence, %  Median [IQR] |  | Adherence, %  Mean (SD) | Adherence, %  Median [IQR] | People with no exercise minutes W13-26, n(%)^c^ |  | | Adherence, %  Mean (SD) | | Adherence, %  Median [IQR] |  |
| NNSW  (n=69) | 53 (41) | 54 [13, 94] | 2 (3) |  | 54 (40) | 56 [14, 95] |  | 33 (42) | 0 [0, 76] | 35 (51) |  | | 66 (37) | | 76 [29, 102] |  |
| MNC  (n=79) | 68 (39) | 74 [36, 99] | 3 (4) |  | 71 (37) | 76 [43, 100] |  | 39 (41) | 31 [0, 78] | 24 (30) |  | | 55 (39) | | 50 [16, 94] |  |
| Austin  (n=36) | 57 (43) | 60 [10, 95] | 3 (8) |  | 63 (42) | 62 [22, 101] |  | 29 (42) | 4 [0, 44] | 10 (28) |  | | 41 (44) | | 24 [3, 80] |  |
| Australia  (n=184) | 60 (41) | 62 [18, 97] | 8 (4) |  | 63 (40) | 66 [22, 97] |  | 35 (42) | 10 [0, 72] | 69 (38) |  | | 55 (40) | | 50 [16, 97] |  |
| Northern England (n=62) | 31 (38) | 9 [0.5, 70] | 13 (21) |  | 40 (39) | 25 [2, 79] |  | 18 (32) | 0 [0, 19] | 41 (66) |  | | 53 (36) | | 53 [15, 85] |  |
| Whole sample (n=246) | 53 (42) | 54 [10, 90] | 21 (9) |  | 58 (41) | 60 [16, 92] |  | 30 (40) | 3 [0, 58] | 110 (45) |  | | 55 (40) | | 52 [16, 104] |  |

^a^Exercise time is progressed in the first 9 weeks. Target dose: week 1-2=40 mins; week 3-4=60 mins; week 5-6=80mins; week 7-8=100 mins and from week 9 onwards=120 mins; adherence is reported as the percent of the prescribed dose achieved.

^b^The whole sample column includes all participants, regardless of adherence

^c^Exercisers are defined as those participants who had exercise minutes recorded in the time frame reported

^c^Initially *StandingTall* did not record exercise minutes if the session was not completed, this was changed on 16 Feb 2021. In Australia, all (n=8) of the people who recorded no exercise minutes from week 1-12 started participating before this date and some (n=3) have acknowledged poor adherence and therefore potentially did undertake some exercise (incomplete sessions). In the UK, 5 of 13 people who recorded no exercise minutes from week 1-12 started participating before this date and one acknowledged poor adherence and therefore potentially did undertake some exercise (incomplete sessions). In Australia, 34 of the 69 (49%) without exercise minutes during the 13-26 week period, finished before 16 February 2021 and therefore may have undertaken some exercise, but if they did not complete the session it would not have been recorded. In the UK, no-one finished before 16 February 2021.

## Supplementary Table 2. Exercise minutes per week by site, country and total

| Recommended dose per week | | 40 mins | | 60 mins | | 80 mins | | 100 mins | | 120 mins | | | | | | | | | | | | | | | | | | Total mins |
| --- | --- | --- | --- | --- | --- | --- | --- | --- | --- | --- | --- | --- | --- | --- | --- | --- | --- | --- | --- | --- | --- | --- | --- | --- | --- | --- | --- | --- |
| Week | | 1 | 2 | 3 | 4 | 5 | 6 | 7 | 8 | 9 | 10 | 11 | 12 | 13 | 14 | 15 | 16 | 17 | 18 | 19 | 20 | 21 | 22 | 23 | 24 | 25 | 26 |  |
| NNSW LHD (n=69) | Mean (SD) | 24 (19) | 32 (23) | 38 (29) | 38 (31) | 44 (39) | 44 (41) | 48 (47) | 45 (49) | 46 (55) | 54 (62) | 46 (58) | 44 (56) | 47 (58) | 41 (58) | 41 (55) | 42 (55) | 43 (59) | 40 (56) | 37 (56) | 38 (54) | 38 (55) | 36 (54) | 37 (54) | 33 (53) | 36 (56) | 41 (58) | 1056 (1122) |
|  | Median [IQR] | 19 [12, 40] | 40 [4, 48] | 44 [0, 65] | 41 [0, 67] | 46 [0, 84] | 56 [0, 85] | 50 [0, 102] | 22 [0, 101] | 0 [0, 113] | 0 [0, 123] | 0 [0, 123] | 0 [0, 121] | 0 [0, 121] | 0 [0, 120] | 0 [0, 112] | 0 [0, 116] | 0 [0, 120] | 0 [0, 112] | 0 [0, 116] | 0 [0, 99] | 0 [0, 97] | 0 [0, 74] | 0 [0, 89] | 0 [0, 72] | 0 [0, 104] | 0 [0, 120] | 428  [86, 2284] |
| MNC LHD (n=79) | Mean (SD) | 23 (20) | 40 (27) | 50 (30) | 51 (36) | 57 (36) | 57 (44) | 65 (49) | 65 (50) | 69 (55) | 70 (57) | 61 (60) | 62 (57) | 57 (60) | 53 (56) | 54 (60) | 54 (58) | 48 (56) | 45 (56) | 47 (55) | 46 (58) | 39 (55) | 41 (55) | 44 (60) | 43 (57) | 42 (61) | 35 (54) | 1318 (1042) |
|  | Median [IQR] | 18 [8, 42] | 44 [17, 64] | 59 [20, 70] | 63 [12, 80] | 68 [23, 86] | 75 [0, 97] | 83 [0, 106] | 76 [0, 107] | 71 [0, 124] | 83 [0, 124] | 52 [0, 124] | 68 [0, 123] | 24 [0, 124] | 33 [0, 122] | 0 [0, 124] | 20 [0, 122] | 22 [0, 119] | 0 [0, 104] | 0 [0, 104] | 0 [0, 122] | 0 [0, 95] | 0 [0, 101] | 0 [0, 113] | 0 [0, 120] | 0 [0, 119] | 0 [0, 91] | 1086 [353, 2213] |
| Austin (n=36) | Mean (SD) | 22 (20) | 35 (26) | 38 (33) | 40 (36) | 51 (43) | 52 (46) | 54 (51) | 56 (55) | 55 (57) | 55 (55) | 53 (55) | 50 (57) | 40 (56) | 43 (57) | 40 (53) | 38 (58) | 39 (57) | 27 (55) | 40 (59) | 34 (54) | 37 (57) | 35 (57) | 30 (51) | 29 (52) | 28 (49) | 26 (48) | 1056 (1082) |
|  | Median [IQR] | 17 [6, 39] | 39 [10, 60] | 32 [0, 68] | 40 [0, 71] | 56 [0, 85] | 57 [0, 99] | 59 [0, 100] | 38 [0, 114] | 28 [0, 122] | 39 [0, 120] | 39 [0, 119] | 33 [0, 93] | 0 [0, 59] | 5 [0, 92] | 5 [0, 83] | 0 [0, 92] | 0 [0, 84] | 0 [0, 66] | 0 [0, 108] | 0 [0, 57] | 0 [0, 80] | 0 [0, 55] | 0 [0, 48] | 0 [0, 33] | 0 [0, 37] | 0 [0, 35] | 664 [86, 1690] |
| Australia (n=184) | Mean (SD) | 23 (19) | 36 (25) | 43 (31) | 44 (35) | 51 (39) | 51 (43) | 57 (49) | 56 (51) | 58 (56) | 60 (59) | 55 (58) | 53 (57) | 50 (59) | 46 (57) | 46 (57) | 46 (57) | 44 (57) | 41 (56) | 42 (56) | 41 (56) | 39 (55) | 38 (55) | 39 (56) | 36 (54) | 37 (57) | 36 (55) | 1168 (1082) |
|  | Median [IQR] | 18 [9, 41] | 41 [12, 52] | 53 [12, 67] | 56 [0, 70] | 61 [0, 84] | 65 [0, 88] | 63 [0, 104] | 52 [0, 105] | 54 [0, 122] | 52 [0, 123] | 33 [0, 123] | 32 [0, 122] | 0 [0, 122] | 0 [0, 119] | 0 [0, 119] | 0 [0, 120] | 0 [0, 115] | 0 [0, 103] | 0 [0, 106] | 0 [0, 103] | 0 [0, 91] | 0 [0, 83] | 0 [0, 103] | 0 [0, 90] | 0 [0, 91] | 0 [0, 83] | 818 [137, 2195] |
| Northern England (n=62) | Mean (SD) | 14 (18) | 18 (24) | 22 (29) | 26 (33) | 29 (40) | 27 (37) | 31 (44) | 29 (44) | 27 (48) | 25 (46) | 25 (44) | 27 (47) | 24 (46) | 27 (49) | 27 (47) | 22 (44) | 22 (42) | 25 (47) | 24 (46) | 21 (44) | 21 (46) | 19 (44) | 19 (43) | 16 (36) | 17 (39) | 16 (37) | 600 (897) |
|  | Median [IQR] | 6 [0, 21] | 0 [0, 44] | 0 [0, 47] | 0 [0, 63] | 0 [0, 70] | 0 [0, 60] | 0 [0, 66] | 0 [0, 67] | 0 [0, 52] | 0 [0, 33] | 0 [0, 35] | 0 [0, 63] | 0 [0, 12] | 0 [0, 45] | 0 [0, 65] | 0 [0, 3] | 0 [0, 12] | 0 [0, 18] | 0 [0, 15] | 0 [0, 0] | 0 [0, 0] | 0 [0, 0] | 0 [0, 0] | 0 [0, 0] | 0 [0, 0] | 0 [0, 0] | 70 [2, 1114] |
| Whole sample | Mean (SD) | 21 (19) | 31 (26) | 38 (32) | 39 (35) | 46 (40) | 45 (43) | 50 (49) | 49 (51) | 50 (56) | 51 (58) | 47 (27) | 47 (56) | 43 (57) | 42 (55) | 41 (55) | 40 (55) | 39 (55) | 37 (54) | 37 (54) | 36 (54) | 34 (53) | 33 (53) | 34 (54) | 31 (51) | 32 (54) | 31 (51) | 1025 (1066) |
|  | Median [IQR] | 13 [3, 34 | 37 [0, 50] | 39 [0, 66] | 42 [0, 68] | 49 [0, 84 | 37 [0, 85] | 51 [0, 103] | 33 [0, 104] | 19 [0, 120] | 20 [0, 122] | 0 [0, 119] | 0 [0, 119] | 0 [0, 122] | 0 [0, 118] | 0 [0, 104] | 0 [0, 107] | 0 [0, 101] | 0 [0, 86] | 0 [0, 76] | 0 [0, 70] | 0 [0, 69] | 0 [0, 64] | 0 [0, 66] | 0 [0, 57] | 0 [0, 64] | 0 [0, 65] | 598 [76, 1869] |

***Note.*** LHD = Local Health District; mins = minutes; MNC = Mid North Coast; NNSW = Northern New South Wales

## **Supplementary Table 3.** Exploring adherence to the *StandingTall* programme

Represented at the participant and health professional/exercise specialist levels.

| **Barriers to adherence** | | |
| --- | --- | --- |
| ***Participant level barriers to adherence*** | | |
| Participants found the repetition of some exercises a disincentive to continue | *It’s a bit repetitive and rigid* | *“doing the same things again and again and again, I just felt a little, ‘oh I’m sick of doing this.’ (5880YJM)* |
|  | *It wasn’t challenging enough and I got bored* | *“I didn’t think it challenged me enough and that was probably why a bit of boredom set in.” (5219FHO)* |
| Self-efficacy impacts on the participants drive to participate | *Finding the cognitive exercises too hard is a barrier to continuing with StandingTall* | *Well, it does put me off. You don’t feel so good about yourself. … Because there’s too much pressure on you. And like I just said, everybody has something wrong with them, you know mentally or physically, that’s why we’re doing this at our age group. We want to feel confident, and we want to feel great and pat ourselves on the back for achieving things. But this is almost like putting me down.” (5346EXY)* |
| Medical issues or more pressing health conditions took priority | *Treatment for medical conditions* | *“I’ve just given it a rest for a couple of weeks because I found it was aggravating my foot a bit, especially during the stepping up and stepping down bit.” (5880YJM)* |
| People have busy lives | *I’m busy and it’s hard to fit it all in* | *I thought, oh, yes, I’ll give that a go. At first, I went up, tried to do the two hours but I couldn’t do it. I only did it in the end to one hour and even that, now, I would struggle to do that because I’ve so many other things to do so that’s why I stopped really. (GM005)* |
|  | *After a full day at work I’m exhausted* | *Well I just thought I’d committed to it to start with and I was making comparison with the exercises I was already doing. And I might have been more motivated had I done it – I was off work for 6 months – so I might have stuck it out for 6 months if I hadn’t been going back to work either. Because now I just think well this is another thing I need to fit into my timetable.” (5141DCN)* |
| Technology has an impact on successful engagement | *You can’t pause a session without it returning to the start* | *Yes, I stopped once and then when I went back to it, I had to start all over again. So, I thought “stuff that”.” (5038VDC)* |
|  | *Technology connectedness was a barrier* | *Well, the catch is that it’s on a tablet. And the wife and I both use the tablet quite often. If she’s got it, I’ll do it later and that later never comes.” (5746CRN)* |
| ***Health professional/exercise specialists level barriers to adherence*** | | |
| Technology has an impact on successful engagement | *Tech barriers reduce confidence in using StandingTall* | *I was turned off by some of the IT things, and I just wanted to hand it over, as soon as I had issues, I just didn’t really wanna deal with it. (VU92)* |
|  | *IT skills of the user were a barrier to progression* | *I guess you want to, firstly, the biggest reason to kind of do these calls is to sort of see why it is that they're not sort of progressing. And one of the main reasons is mainly technology issues. And it's just that they may not have wanted to communicate with me about the troubles that they're having. So, when I eventually make contact with them, I sort of give them a solution to that issue, and then from there it kind of enables them to sort of progress a little bit further. But often if they’re having issues with the program, they often don't tell me until I get in touch with them. (QP26)* |
| Medical issues or more pressing health conditions took priority | *Patient health influenced compliance* | *So there was one that there were a lot of mood depression issues that were contributing. So there was some external factors to just the program itself. There was one that definitely went through the full six months. And I just can't think of the other one who I had. But yeah, I mean, that's, that's a problem we have with a lot of clients and compliance with exercise long-term, unfortunately. (NC34)* |
|  | *Other health issues took priority* | *The other one was with the participants we deal with, their ability to do a regular activity programme, because they’ve got health conditions that stop them being active on a regular basis, they were dropping out like flies because something was coming up and, okay, they got a bout of cold or a bad knee or bad back and then they stopped, and then to get back into it, they have other issues, so other priorities, so it was very tricky for them to keep up with it. (GG2 AG)* |
| The research specific components were a barrier to adherence | *The research component overshadowed the enjoyment of using the program* | *there was all this red tape and surveys and the email or the surveys go but they never worked or people found them like taking over their accounts … so some of them were wondering, what’s going on with my email? My email’s gone now. This thing’s taken over. I lost a few people with that, thinking that it’s, you know… No, I’m not having it. Then I was like, oh, well…and then [] was like, we can send them the survey, you know, via their email. (GG3)* |
| People have busy lives | *Time poor; finding the time to do another thing* | *oh, it’s another thing, then, that I’ve got to find time to do. (TT1)* |
| Social connection is a desired element of an exercise program | *The social aspect to exercise is important for long term participation* | *they weren't interested just by, you know, showing them the technology in the program. They just didn't feel that was what they wanted. They needed more of the social aspect of their exercise, as opposed to doing it at home, because they just didn't feel like they have the motivation to, or the discipline to do it themselves. So the ones I did refer were ones that went on with it, and did find it useful for the period of time that they were doing it. There was no, despite all the follow up phone calls, there was no long-term compliance with the program. (NC34)* |

## Supplementary Table 4. Referral source and uptake for Australian sites

| Referral pathway, n(%) | NNSW | |  | MNC | |  | Austin | |  | Whole sample | |
| --- | --- | --- | --- | --- | --- | --- | --- | --- | --- | --- | --- |
|  | **Referred to study, n=176** | **Enrolled in study, n=69** |  | **Referred to study, n=181** | **Enrolled in study, n=79** |  | **Referred to study, n=88** | **Enrolled in study, n=36** |  | **Referred to study, n=445** | **Enrolled in study, n=184** |
| Community/ self-referrals | 81 (46) | 32 (40) |  | 44 (24) | 33 (75) |  | 14 (16) | 7 (50) |  | 139 (31) | 72 (39) |
| Health promotion programme | 53 (30) | 21 (40) |  | 38 (21) | 11 (29) |  | 0 | N/A |  | 91 (20) | 32 (17) |
| Health service clinicians offering inpatient services | 0 (0) | N/A |  | 0 (0) | N/A |  | 5 (6) | 0 (0) |  | 5 (1) | 0 (0) |
| Outpatient/ community-based clinical services | 37 (21) | 11 (30) |  | 21 (12) | 9 (43) |  | 67 (76) | 28 (42) |  | 125 (28) | 48 (26) |
| Private practice clinicians | 5 (3) | 5 (100) |  | 59 (33) | 19 (32) |  | 2 (2) | 1 (50) |  | 66 (15) | 25 (14) |
| Community service agency | 0 (0) | N/A |  | 19 (11) | 7 (37) |  | 0 | N/A |  | 19 (4) | 7 (4) |

*Note*. MNC = Mid North Coast; NNSW = Northern New South Wales

## Supplementary Table 5. Exploring the uptake and adoption of the *StandingTall* programme to provide direct guidance for programme scale up

Represented at the participant, health professional/exercise specialist, health service manager and study staff levels.

| **Barriers** | | |
| --- | --- | --- |
| ***Participant level barriers to uptake and adoption*** | | |
| The technology is either a perceived or actual barrier to overcome | *StandingTall is not compatible with android devices* | *Oh, I’m pretty tech savvy, not very happy with the App, it’s not compatible with Android devices. So, I cannot use it on either my mobile phone or my tablet, I’m stuck with my laptop, so, that’s a pretty major issue I think. (5481GYL)* |
| Despite its flexibility, there are still some barriers to exercise | *It’s preventing people from participating in their normal exercise routine* | *one negative with it is that I’m not doing as much tai chi as I normally do. Because it’s taking 2 hours out of my week, which I may have gone and done a bit more tai chi. (512UKL)* |
|  | *Not knowing why you are doing some exercises; there’s no way to check* | *this is what I thought, I didn't have anyone to look at how I was doing things as to whether like, you know with like a physio background I think I was doing things okay, but you don't know with your own body. And I wasn’t anywhere where there was a mirror, so I couldn’t look. (5134APM)* |
|  | *It needs additional equipment that is hard to source* | *The equipment is a problem. It depends on how your house is set out really. ... The one big that I miss, and I’ve not found anything that actually meets its requirements short of having one specially made, is a step that’s the right size. Even when I’ve gone back to the gym, you can get steps that you can step over, but they’re not good for the sideways because they’re too wide (W203)* |
|  | *It’s missing the advice you get from your health professional/exercise specialist* | *I mean that's what's missing, there's nobody to advise you really. I know you can ring your trainer up, or it's…you know, that person is not seeing you, they're not seeing how you're performing. You know, perhaps maybe you're doing something wrong. You just don’t know. Even though you’ve looked at the, you know, instructions, read the instructions you might just be doing one thing that is wrong, or making it harder, you know? (W024)* |
| Tailoring it to the right patient | *The programme needs to be targeted to the exercises the participant finds challenging or can’t do* | *the programme doesn't seem to be totally targeted at what I can't do. So, you know, I probably should, when I’m not doing the programme, should do some more of those things that I can't do to practice. But that's why I think the programme should be a little bit more targeted. (5072PCH)* |
| ***Health professional/exercise specialist level barriers to uptake and adoption*** | | |
| The technology is either a perceived or actual barrier to overcome | *Assumption you have to be young and tech savvy to use StandingTall* | *You have to be quite selective with your patients as well. They’d have to be quite high functioning and computer savvy. Which means that a lot of our population that we see in public health are your elderly, or your patients with a lot of co-morbidities as well. Which makes it a challenge for something like the StandingTall App. But there are exceptions to the rules, where you’ve got some people in their early 90’s who do know one end of a tablet from another. But you know, for the majority of that age group, I think you know, they struggle a bit. (FJ23)* |
|  | *Using technology can scare some patients, but can be overcome* | *That might scare them in the first instance, once you start talking about technology, ‘oh, no, no, no, no I can't do that.’ We do get a bit of that. (GH61)* |
|  | *Technical literacy of the user is a barrier* | *So, I think for most of those who haven’t done it, who haven’t done it at all, was the IT problem, getting it to work or to make it work, not because it wasn’t working, but because of their limited ability to deal with IT and computers and tablet and stuff like that, that was one of the big problems. (GG2)* |
|  | *It relies on stable internet* | *Yes, internet was very unpredictable and clients … That was the other thing too, you know, depending… I don’t know if it was the internet or whatever. You know when they would do the demo and either have it work or demo, I mean like it was just…it took ages, buffered and buffered and buffered. (GG3)* |
| How it is resourced will impact on uptake | *People need training to have the confidence to use the back-end of the app* | *For me, personally, actually, yeah. It was the backend of going into the back…I didn’t feel confident with that. I did feel out of my depth. Again, because it was something that was passed on to me because somebody else was moving on, I just felt that I didn’t really know what I was doing with it. (WW2)* |
|  | *Resourcing: the need to provide support outside of the research setting* | *The programme, the programme will work, the programme will be successful, but it will only be successful if you’ve got the right people at the other end that are monitoring and managing it. (FF1)* |
| Work demands and other alternatives will impact on uptake | *Competition from other physio apps* | *I suppose my point of view, I mean, is that the main thing I had with StandingTall was that it was a bit fiddly to get around, if that makes sense. I'll be honest, there's so many apps out there. There’s lots of apps out there that you can do, they'll tell you what exercises you can do that's a lot more simpler to follow. I thought StandingTall was actually quite difficult to set up initially. But once you set it up, then it became okay. Yeah, just the whole set-up was quite tricky. Mainly, that's because it was doing it as a research kind of thing. So there's so many apps on the app store that you can get to exercise that, you know, they tell you what exercise to do. And yeah, and then they mark it for you. So with this as well, but I'm not sure why StandingTall, it took a lot more effort to register and to actually fiddle with before we actually get started, which was a bit annoying. (SA78)* |
|  | *Clinicians are time poor and it impacts on workload* | *Yeah, time consuming, and it just requires me to problem solve and do IT stuff which I just didn’t view as my core business. (VU92)* |
| Tailoring it to the right patient | *Having the right patient* | *I think we really need to be specific with choosing the right patient for it. The unfortunate thing with falls is that you really want to improve someone's balance and so forth. You do have to challenge him. So yeah, it’s if, safety wise, that's what you're compromising. Especially if you're not there to assist. So, again, we come back to choosing the right project, making sure that they're competently sensible enough to take on the task at hand. (SA78)* |
| ***Health service manager/stakeholder level barriers to uptake and adoption*** | | |
| The technology is either a perceived or actual barrier to overcome | *Access to Wi-Fi* | *I just thought of another thing too. One of the things that would be a barrier, and sort of has been a bit, is the wi-fi in the hospitals. The hospitals have a very low firewall threshold. It’s not a fire threshold but then they’re really tight on IT. One of the issues for us will be whether staff have access to wi-fi to show someone how the App works. If they’re showing someone on the participant’s own iPad, can the participant access wi-fi to go through it with that, you know that sort of stuff. (LK38)* |
| Upstream determinants will influence uptake | *No Federal level approach to falls prevention* | *no falls prevention policy at the federal level either and therefore no real driver for investment in Standing Tall (DG19)* |
|  | *No State level approach to falls prevention* | *Falls prevention is not currently a health prevention policy and you need policy to ensure a health issue is funded and a focus of work. There is no NSW Government policy in place for falls prevention and it is not a core focus of their work. (DG19)* |
|  | *Prevention efforts are focussed on healthy eating and being active* | *they have taken a strategic decision to focus on healthy eating and obesity and being active. Focus on changing individual behaviour e.g. via telephone counselling. In terms of falls they have pulled out from doing any work in that area. (VX45)* |
|  | *Falls prevention is decentralised to each LHD* | *basically the Ministry of Health is leaving it to the Local Health Districts to ways of coming up with how they’re going to spend their money. (RD52)* |
| Despite its flexibility, there are still some barriers to exercise | *Clinicians are time poor and it needs a champion to support it* | *There has to be something in it for them and/or they see really big benefits for their patients so that they can’t no help share this great new service. That would probably be the other barrier for me, is that we have very time poor clinicians up here. I think it’s everywhere. (KP27)* |
|  | *Alternatives for accessing technology* | *Of course, the other thing, and I know it’s not huge because a lot of older people are familiar with technology, but of course, there are people in the targeted group that don’t have laptops or tablets and probably never will. We’d have to think of another way of accessing them. (RD52)* |
|  | *Equity and the digital divide* | *That suits some people and you’re really narrowing down and you're probably creating quite an equity issue in terms of the people that suit that are probably your more educated, more well off people at the first place. (EY63)* |
| How it is resourced will impact on uptake | *COVID-19* | *I just don't think we've come up with a solution about how to roll it out without, you know, without funding somebody to do it. I don't think, I mean, and whether that's, as I said, I'm not sure how much of that’s due to COVID. You know, if we had had a completely clear run, would we have had more engagement with our LHD staff? Would we have had more engagement with private practitioners? Would we have been able to kind of convince them that they could do it? I don't know. It's still up in the air, I think. (EY63)* |
|  | *Training and who is paying for the initial assessment* | *The main thing if people who are trained up in how to set someone up, like an ET and there’s a way of paying them for that one off session, through a package or because they already work for an organisation, then it would be fine. But that would probably be the issue of who’s going to pay for that initial assessment, initial set-up process when it’s not done by us and therefore not free. It has to come from somewhere. As I said, we’ve only just started to touch on this and I think it would be quite highly utilised once people realised how good it was. (LK38)* |
|  | *There is a need to buy other equipment* | *In terms of barriers, we’ve bought the foam and the stepper, because that in itself would have been about $50 in costs. (LK38)* |
| ***Study staff level barriers to uptake and adoption*** | | |
| The technology is either a perceived or actual barrier to overcome | *Having the right technology* | *... early on we had the option where we could use it in Apple device but we couldn’t use it in a Windows device or an Android. Imagine the conversation you’d have when you go, “What sort of device have you got?” “I don’t know”. If you’ve got this we can use it, but if you’ve got that we can’t. ... (MP87)* |
| Despite its flexibility, there are still some barriers to exercise | *It needs someone to provide guidance to clinicians* | *Clinicians don’t quite understand at what level this programme is useful for. (MP87)* |
| How it is resources will impact on uptake | *Not knowing it was an effective programme* | *… you haven’t got the evidence to say what this does. … Because clinicians will say this is the evidence, this thing here I’m going to strongly back this because it’s got really strong evidence done in a really controlled way that this program here suits this particular group of people, and I’m going to be able to offer this for this particular group of people. (MP87)* |
|  | *Hospital departments often have a siloed focus which prevents change* | *I think it’s a practice change issue. People taking time to change practice. There’s also some systems which are all these little silos that aren’t working together. (MP87)* |
|  | *Resourcing: the need to provide support outside of the research setting* | *The first question you’re asked when we want you to do this, setting up people and doing this for your clients. Sometimes it’s like “Is there any funding that goes with this?” (ZG40)* |
| Work demands and other alternatives will impact on uptake | *Competition from other physio apps* | *The other thing is that exercise physiologist at [suburb], he was talking about he uses a system called [name of exercise app] and we’re going to have a look at that and see how that works. (ZG40)* |
|  | *Clinicians are time poor and it impacts on workload* | *They’re a busy clinician... it’s great that you can remote in to have a look at what people are doing and all that, but it does take time to understand what the data actually means and there’s clunky spreadsheets and all that. They’re not intuitively quick and easy to look at. The clinician’s got the ability to have a look on the person’s iPad and just get a quick idea going, “Okay, yes you’re doing a certain amount of exercise and this is the level of exercise”. But then if you want to go more detailed, you have to go into the backend, to the content management system, and that is just really hard to get your head around. I’ve looked at a lot and I’m very good with Excel spreadsheets and graphs and all the other, but it’s still taking me time to understand this really complex laborious links of information that clinicians are probably not going to spend the time to really understand it, to get comfortable with it. (MP87)* |
|  | *COVID-19 made it difficult to build relationships, which impacted on uptake* | *The team found it difficult to build relationships with exercise instructors and speculated that the reason being COVID restrictions prevented the team from meeting with instructors in person and that this impacted on recruitment rates (ET76).* |
| Tailoring it to the right patient | *COVID-19 created a cohort with complex health needs, impacting on perceived suitability* | *[Health professionals/exercise specialists] Expect their participant cohort to be more fragile and unwell than [other] participants. Physios report that rehab services are seeing people with more complex needs and deconditioned because of the lockdowns. (ET76)* |
|  | *The research eligibility requirements were restrictive* | *We also have had a lot of people who have come into this program who may not be suitable. Because of language issues or they might not have IT access. They’ve also have other things, complicating things. So they might have got some cognitive problems. They might also have a neurological condition so they might have MS or Parkinson’s Disease or something like that, they’re not eligible. So there’s a whole group of people who may benefit from Standing Tall, but they’re not actually meeting the selection criteria. (MP87)* |
| **Facilitators** | | |
| ***Participant level facilitators of uptake and adoption*** | | |
| *StandingTall* (design and delivery) lowers barriers to uptake | *The delivery mode lowers barriers to participation* | *look I just think that the programme is suited for people who, particularly who can’t you know, have to go by public transport to get to- so if you had to go to the hospital to do the exercise. (5248KHQ)* |
|  | *Using the App makes you more engaged* | *I think just using the App. Because a) you’re more engaged. So, I think, yeah, that engagement of having the App and having that time factor. It worked for me, I liked that. (5134APM)* |
|  | *StandingTall is tailored to suit your needs and lifestyle* | *But as I said to you, the reason I’m such a fan of it is because it suits my lifestyle. (5686AJ)* |
|  | *The programme can run on a PC, which helps people with poor eyesight* | *And I said, can I have it on the PC, because of my eyesight? And he said, yeah, of course you can. So, I knew I could do that with WhatsApp, and that’s what I’ve done with StandingTall, I’ve put it on my PC, and it’s better for me… …because if I had an iPad or was trying to do it from a phone, my eyesight isn’t good enough to see that, I’d have to be really on top of it. And I do struggle a little bit, at the moment, because I’m still waiting for some eye surgery, I do struggle a little bit, you know ... (MM175)* |
|  | *The way it is delivered is better than alternatives* | *… it's a programme that’s better than giving them a sheet with exercises on, because they won't read that anyway. But if they’ve got this app then they might get people back into, you know, rehabilitation sort of thing. (W024)* |
| Patient-centred outcomes are important for driving uptake | *I feel better when I do the exercises* | *I just felt generally more stable on my feet and just physically better, physically more energised by exercising. (5481GYL)* |
|  | *You don’t need to spend a lot of money to do StandingTall* | *look, I’ve been really happy with the way it’s going. And I just love the fact that the equipment I need, I’ve got it at home, and I didn’t have to go out and spend a lot of money on it. (5248KHQ)* |
|  | *Concern about future falls and poor health motivated participation* | *... it’s been a factor that I’ve been thinking about really. So yeah, I would say over the last 12 months, really. And in that time my sister was getting worse and worse, and it was…in the back of my mind, I don’t really want to finish up the same as she is ... (W010)* |
|  | *Physical activity is important for my health (condition)* | *I like to think in fact my consultant at said that it's the exercise programme and what I do this is keeping me in a virtually upright position because I have spinal [condition] and the amount that I do is actually helping to strengthen my muscles which keep the spine in a good position which is important for me because I certainly don’t want to face at this time surgery. (T001)* |
| Commitment, obligation and goal setting drive uptake | *I felt obligated to a research study* | *Well, I thought I was doing it to help some sort of programme or research. (5096NOX)* |
|  | *Making a promise to oneself that the goal will always be met* | *the thing I find that keeps me on track is that I made the promise to myself that the goal will always be met. (5686EAJ)* |
| Support and incentives are still important drivers of uptake | *Access for the physio to be able to see their clients progress* | *… as I'm going to go to her, which I did a few times, and ask for help, she needs to know what exercise I'm talking about, and why it may be difficult. (5346EXY)* |
|  | *Generating weekly progress emails, so I can see my progress* | *perhaps just, again, just a prompt that says the last week, recognising the fact what’s done, what progress is made and see what goals did you set yourself and your goals for next week. Maybe that’s the kick in the backside I need to get me committed, I don’t know. (5219FHO)* |
| ***Health professional/exercise specialist level facilitators of uptake and adoption*** | | |
| *StandingTall* (design and delivery) lowers barriers to uptake | *COVID-19 removed barriers to eHealth* | *a lot of our clients, particularly with COVID, now are wanting to stay doing things at home, as opposed to coming in, doing things in groups or in community environments. So you know, I think that having as many options available, is, is more useful for us than the limited options that we had previously. Because I think having that visual feedback, having the flexibility, having the ability to progress and regress as needed, that they can self-report is really handy. (NC34)* |
| *StandingTall* fulfills a need and there is support to prevent falls | *It complements clinical care* | *Yeah, I think it's really, for the [patients] who are coming into the clinic for exercise, just implementing that. Because they’re just working on the balance with StandingTall. It's easy for me just to then focus on strength or other things because I know that they are already doing that. But yeah, I can see their balance improving. (HX54)* |
|  | *Action to prevent falls is what we should be doing* | *My view on it is that anything that we can be doing to prevent falls is something we should be doing. (TS65)* |
|  | *Fulfils an unmet clinical need* | *I was actually quite excited about this StandingTall program. Obviously, we see a whole range of older folk, some that come in with specific mobility issues and falls, but others who come in with other things who are still quite well, but just, you know, they'll say, ‘oh, getting old’s no fun, I’m just not as good on my feet as I used to be.’ And so, I feel like it's a great option for sort of people at a slightly higher level to the ones we would refer, say, to the falls and balance clinic, or the ones that would have to come in and be admitted. Yeah, so I feel it's a really good option sort of to offer people for that sort of middle, middle group of people who are just starting to slow down and struggle a little bit, but don't have, you know, serious issues with their mobility and their function. (LM13)* |
|  | *Evidence-based, high standard programme that offers value* | *I thought it was really novel and also had good results, so that’s why I was quite interested in it. And when [IO] was saying there’s the library of exercises was quite enormous, like from memory it’s like 6,000 different exercises. I thought “aw, that’ll be really useful for patients”, at different levels as well. So, yeah, I quite liked the fact that it could progress patients, or the other way around as well. (KN73)* |
| Patient-centred outcomes are important for driving uptake | *It’s user friendly and benefits the patient* | *it's very user-friendly. I think, as more people become more comfortable with technology, it's going to be easier to get people on board. (NC34)* |
| ***Health service manager/stakeholder level facilitators of uptake*** | | |
| *StandingTall* fulfills a need and there is support to prevent falls | *Keen to utilise technology to improve health* | *but generally our health district is very keen to utilise technology. Our region is geographically dispersed and does contain quite rural places and we one of the fastest largest growing ageing population in Australia, particularly our [ ] Local Government Area. So the fact that our population demographics and geography and our goals around better utilising around technology to provide care to people and keep them out of hospital really fit neatly with the Standing Tall programme. (KP27)* |
| Support and incentives are still important drivers of uptake | *Incentives to use it – money, patient health outcomes, quality accreditation need to be considered* | *I’ve tried to sell programmes to general practices before and I guess the things that I would try and sell, I guess money talks. If there’s a financial incentive for them to recruit and onboard participants, then that would certainly help. I think promoting the fact that their involvement in this programme helps them achieve some of their other quality improvement, or quality assurance processes. So, for GPs, I’m often trying to talk about how promoting this Health Promotion programme would achieve X, Y and Z in the quality accreditation standards. Obviously, people will do it to benefit their patients, so having some really good support around selling the programme to their patients, why it’s beneficial, what are the improvement benefits, helping the clinicians really understand that, and make it easy for them to sell to patients health. So they’re probably the three big ones. Good for the patients, good for their back pocket, and provide some evidence to support them to meet their quality accreditation or quality improvement standard. (KP27)* |
|  | *There needs to be alternatives for accessing the technology* | *the easier you can make it for these people, the more likely it is to happen, so whether there’s a lending library, or what there is, with the physios. And of course, storage is an issue, storage of these things, because they do take up room. (RD52)* |
| ***Study staff level facilitators of uptake and adoption*** | | |
| *StandingTall* fulfills a need and there is support to prevent falls | *It complements clinical care and can be embedded into procedures and practice* | *my intention was very much on making this the real world and being embedded into existing services and practices. I still feel like that. I still feel like that strongly, that we really should be trying to do that, so this is a sustainable thing that doesn’t go away when you have extra people like implementation officers or supporting team members to do it. It has to be embedded. (UD15)* |
|  | *Fulfils an unmet clinical need* | *our Health Promotion team for [geographical area], we’ve got a Falls Prevention coordinator that works within the Health Promotion unit. In that team, the Falls Prevention team sits within the Healthy Lifestyle team of the Health Promotion unit. We’ve got a lot of Healthy Ageing focus. Falls Prevention sits in there and StandingTall sits in there as well. Obviously, they’ve got things where they’re supporting group classes like Stepping On. StandingTall seems as something that’s ongoing for people to continue. Like they’ll do their Stepping On, and they’ll get a lot out of that, but StandingTall is that next level of falls and balance exercises they can do, balance exercises. So it’s the next level. I think it’s perceived as opening more avenues for people to participate in falls prevention programmes. As I said, supporting keeping people active and prevent falls in the first place, is where the focus is with the Health Promotion unit. The StandingTall balance programme does fit in with that model. (ZG40)* |
|  | *Evidence-based, high standard programme that offers value* | *It is a preventative approach. It’s evidence based. There’s not too many Apps that are. (ZG40)* |
|  | *There is community interest in fall prevention* | *… there is a lot of interest in falls prevention from the community, particularly the older population. People want to hear about it, how to prevent it, what programmes are available. … (ZG40)* |
| Support & incentives are still important drivers of uptake | *Having a central hub to help clients with low IT literacy* | *There does need to be a supportive tech person to help people with all the tech stuff because it is tech. And then you’ve got the wi-fi part as well. (MP87)* |
|  | *It is user friendly and benefits the patient* | *As a clinician, they might have a thousand things to now choose from, and they have to work out which ones. Most people will go back to something that they feel is comfortable which is a reasonably good product, and that they’ve probably had feedback from clients or patients that it’s a user friendly valuable thing. I think they’re strong incentives for clinicians. If someone gives them good feedback about a particular device or they might see someone do the program using this Standing Tall, and then they came back and they saw how much they benefited, those sort of things are also really informative, which people would be more likely to promote the product and support it and embrace it (MP87).* |
|  | *Clear definition of who the target audience is* | *I think probably being really clear on who it actually is suitable for because you’ll find people might pick it up and it’s not really suitable for them. There’s going to be a lot of people who are high risk, so I think they need to be really clear saying, “This is the type of person who it’s relevant for and this is not who it’s relevant for” (MP87).* |
| Understanding the health environment/context may drive uptake | *Understanding the health system will help direct uptake* | *These services are funded by [government] or public health units within local councils but delivered by contractors. The services are commissioned and operate under [government] policy guidelines. Offer group-based exercise programs. Also offer a paper-based self-directed program that patients can use at home. The team hypothesised that StandingTall might offer an alternative to this programme. (ET76)* |
|  | *It also needs top-down support at a local level* | *Okay. Needs management support at a high level. They need to be invested. They need to recognise that, as I said, it’ll save them money in the long run. (ZG40)* |
|  | *Identifying drivers of uptake such as cost-effectiveness, prevention and health promotion will aid implementation efforts* | *All the hospitals have got their own little falls – obviously, they focus on Falls Prevention. They were looking at more how they’re going to use it as a preventative approach once a person’s left hospital and not just how do you prevent falls while people are in the hospital. It could be embedded through that way. The Health system is seen just not, again, as I said, preventing falls in the hospital but in the home. Health Promotion definitely sits well and is received there. Embedded through promoting and keeping to continue to promote falls prevention through the Health Promotion unit, something that you can offer to people. I think that the dollar value gets pointed out. (ZG40)* |
|  | *Developing a programme that is fit for purpose is imperative to successful uptake* | *Key point is the team believe that StandingTall is a hybrid approach; something in between a tool for clinicians and patient self-directed programme. This means compromises and that StandingTall is not completely fit for purpose for either uses. (ET76)* |

## Supplementary Table 6. Exploring the acceptability (i.e. do they like it) of the *StandingTall* programme to provide direct guidance for programme scale up

Represented at the participant, health professional/exercise specialist, health service manager and study staff levels.

| **Barriers** | | |
| --- | --- | --- |
| ***Participant level barriers to acceptability*** | | |
| The programme does not suit every person’s needs | *StandingTall does not suit every older person* | *I’d say it’s now, is has to be, oh how long do you reckon, about five months? Four to five months, I’ve been doing these exercises. (person in background responding). Yeah, I started in November. I realise that for the right people, this would be so beneficial, for the people sitting down in the lounges and not doing much, which I know that in my age category there was a few of us. But I’ve always, with my job and everything I’ve always been pretty active anyway. And we live in a house that’s got 50 stairs in it. (509EVQ)* |
|  | *It’s not a full body exercise programme* | *Not just the core, you actually got to work on the upper body as well. So you’d like a bit of strength in your arms to support you. I just, yeah, I think it just really needs to do all-over exercise for your balance. I think, and like, I'm not talking about aerobics. I'm just talking even like, because I do, some of the work I do in the morning is Pilates and I think that, I would find more beneficial an easy Pilates thing to work up to, you know ... (5094EVQ)* |
|  | *I’m lazy and need something to push me* | *I’m too lazy. … Yeah, I didn’t have the energy. You know, I need something to push me, because I’d rather sit and watch television. (5746CRN)* |
|  | *It was missing the incentive and social elements from face to face or group classes* | *Well, yeah, because that’s just your motivation, you know, you gotta go to the class. … there’s also a social aspect, as well. So, we usually get some coffee afterwards and put on all the weight we just lost. (5038VDC)* |
| While initially the thrill of the new drove acceptance, some of the exercises presented issues to long term acceptability | *Not being able to balance well made me feel like the programme wasn’t helping* | *It put me off a bit because I felt a little silly because you know, it wasn’t helping me balance, it was causing me to become unbalanced. (5746CRN)* |
| ***Health professional/exercise specialist level barriers to acceptability*** | | |
| The programme does not suit every person’s needs | *It can get repetitive* | *But I guess our general reports were that it gets a bit repetitive. Like the exercises are repetitive and things like that. So, I don't know how sustainable it would be for those people that are sort of keen for a challenge. (HV49)* |
|  | *The research component overshadowed the enjoyment of using the programme* | *That was good but then because of the research, there was all this red tape and surveys and the email or the surveys ... we can send them the survey, you know, via their email. (GG3)* |
| For some, the technology is a difficult barrier to overcome | *Time consuming, a session took longer than you wanted* | *another (person) left because of time and I could understand if you were saying, right, I’ll do ten minutes today, really it was sort of 20-25 minutes. So I could understand it was really time-consuming for people, and I think they all felt the same, they all could have got through the exercises a lot quicker had the programme been able to allow that. So yeah, it was…especially one lady, she said she found it – and I think it’s a great programme, I think it just needed speeding up a little bit or you’d be able to manoeuvre round it a little bit better. (WW1)* |
| While initially the thrill of the new drove acceptance, some of the exercises presented issues to long term acceptability | *Patient compliance – fun at first, but interest drops away* | *The other thing I was always sceptical of is obviously compliance. Because I'm not sure, but for me, anytime you get new kind of toys you're quite excited with or new kind of stuff, but then it’s how long will that actually last for. So that was always something that I had on the back of my mind, is it really gonna work and so forth. (SA78)* |
|  | *Doing the exercises caused the patient to experience joint pain* | *One lady actually, also from the stepping on group, felt that, so this was the complete opposite to the one that I just told you about. She felt her knees, she was having problems her knees which she didn't have before, and she put it to the step. She thought it was stepping up and down off the step that was given her problems with her knee, and she was from the stepping on group as well. (GE84)* |
| Users need to be supported to remain engaged over the long term | *Users lost motivation and found the set up boring* | *keeping the patients motivated during the set-up as well was quite hard, because, you do…some of the exercises, like the 10-second balances and that, you kind of do six goes of it and then move on to another variation of it. Whereas, they are used to doing two lots of an exercise with an elastic band, say. So, they move on and there’s quite…there’s a bit of variety. But I think there was a lot of repetition involved, and I think it got a bit frustrating for some of them. (KK1)* |
|  | *Patients need to be supported to stay on track* | *So for the people who have towards the start that have dropped out, I think some of them found it boring and hard to stay motivated, and maybe other things are more important to them. And they just sort of stopped going because they were really just relying on themselves, rather than me turning up to take them through their exercises. (HX54)* |
| ***Health service manager level barriers to acceptability*** | | |
| The programme does not suit every person’s needs | *It needs to consider diversity in age and CALD communities* | *so besides the actual teething issues of the programme itself, our cohort of patients, older, non-English speaking, not very, often not very tech savvy, said, I think quite tricky. (AC12)* |
| ***Study staff level barriers to acceptability*** | | |
| Users need to be supported to remain engaged over the long term | *The expectations about set up need to be clear, it is too long* | *Improvements: make clear up front how long it takes to do assessment and set up. (ET76)* |
| The delivery and design of the programme is critical to its appeal | *The programme needs to be perceived as including a strength component* | *Physios would want strength-based activities added (ET76).* |
|  | *Health professionals/exercise specialists want to be able to adapt the exercise programme* | *Clinicians wanted more control of StandingTall, to be able to adapt for a patient for example, delete and add exercises. …To be taken up by physios they would need the ability to adapt/direct the exercise program (ET76).* |
|  | *Training needs to focus on the back-end of the app and building confidence to use the programme* | *[The] content management system [is] not used by clinicians/exercise instructors. Exercise instructors were not confident about using even after online training in its use (ET76).* |
| **Facilitators** | | |
| ***Participant level facilitators of acceptability*** | | |
| It’s improving my health, confidence and awareness | *It’s improving my health* | *I’m grateful, I’m grateful to have it. I think it’s a wonderful thing, because I know how important this sort of thing is to do. … I think that it’s given me more energy, I used to have a sleep every afternoon, and I don’t have that sleep and I feel that I can get out into the garden and do more. (5248KHQ)* |
|  | *Builds awareness; the need to be aware of your body and surrounds* | *I think that's what I hoped it would do. And just heighten my awareness of what I was doing with my feet and yeah, learning how I was putting my feet in sort of you know, unusual positions and so forth, that I could manage that without it making me feel all wobbly. It definitely gave me more confidence, I think. (5457KNH)* |
|  | *Improves confidence* | *Plus, I’m getting more confident, stepping up and down and things like that. Being more aware of when I stepped down outside of the pavement onto the road, and just little things like that. (5346EXY)* |
| The programme is enjoyable and offers more than just physical activity to the user | *Highlights what they need to work on or improve* | *I haven’t had any negative effects. I think what it has done, is it’s thrown up to me, some problems with my feet, which I wasn’t really aware of, which I’m tackling, so that’s another reason why I think I’m standing better, … (MH105)* |
|  | *It’s easy to use and I love it* | *I like the app, I found it easy to use. Once you’ve done one, it’s easy to use. I absolutely love the dartboard and the grid exercises, ‘cause to me, it’s a bit like doing dance lessons, footprints going all over, I love it, love it. (MH105)* |
| The delivery and design of the programme is critical to its appeal | *Being challenged is critical to enjoyment* | *Well for me it’s the ones that challenge me most, the standing on one leg and closing my eyes, they ones I can’t do easily that I find the most enjoyable ’cause they’re the ones I really think are really helping me with my balance. Because the other ones that are just too easy, it’s like going through rote and you just don’t enjoy doing something that doesn’t challenge you. (W113 & W012)* |
|  | *It’s flexible; you can choose when and where to do it* | *And [StandingTall] gave me the freedom to be able to do it whether I was here or whether I was over in [another city] seeing their granddaughters or whether I was down [at holiday house] where we’re going to retire. So, it gave me all that freedom, so it had a lot of benefits to it. (5219FHO)* |
|  | *The delivery mode lowers barriers to participation* | *I just think that the programme is suited for people who, particularly who can’t you know, have to go by public transport to get to- so if you had to go to the hospital to do the exercise (5248KHQ)* |
| ***Health professional/exercise specialist level facilitators of acceptability*** | | |
| It’s improving my health, confidence and awareness | *Patient’s reported improvements in their balance and strength, and they were more confident* | *the feedback from a couple of them, that they certainly felt a noticeable improvement in their balance and their strength. And then I think, overall, they were more confident as well. (TS65)* |
| The programme is enjoyable and offers more than just physical activity to the user | *Impressive to use; it’s more than just a physical activity programme* | *when I heard about it, I thought, ‘Oh, yes, this looks a really good idea from a balance perspective’. When I used it, you know, with the assistance of [exercise specialist], I thought ‘it's absolutely sensational from a cognitive point of view, as well as the physical balance point of view.’ So I, you know, became a lot more impressed with it when I used it, moreso than hearing about it. When I heard about it first, I was just thinking of it as a physical programme. When I actually used it, you know, the concepts of memory and following instructions and thinking about how exercises, instructions are followed through. It just had so many more advantages. (DY42)* |
|  | *The programme is comprehensive* | *I felt that the programme had good features, and the programme itself is a good mixture of exercises, balance and strength and overall, I’m really, I really think it’s a great programme (GE84)* |
|  | *Patients were enthusiastic about using StandingTall* | *One in particular was really motivated and he was really keen to do the program. And when I was monitoring it, yeah, he was, he was like religiously, whatever the, I think it’s 40 minutes to start off with and then it increased incrementally then it will ramp up to the next. So, he was he was really good. (W113 & W012)* |
| The delivery and design of the programme is critical to its appeal | *The structured, interactive and progressively challenging nature of the programme was appealing* | *That's the patient experience of the App, I haven’t seen an App before that does their progressions like that, most of them are based on the number of times you've completed an activity successfully or something like that. Or just structured to go from week one you do this to week two, you do this without considering the individual. (XA25)* |
|  | *The safe and progressive nature of the programme means patients didn’t need a lot of assistance* | *It's very, very good. It's very, very good. Like, I say, to my patients, you have to read, read the statements and select the right one. And I think that's another thing that was a positive for me was the rating of how stable from unstable to very stable, that scale was well articulated so patients could choose the right one. And so I thought that was very good. So, with that enrolment, once they get that concept, and they understand that, they can, you know, I found that the progressions built into the system were appropriate and therefore would be confident to let you know, after a couple of weeks check in, let them go on for their six months, 12 months, however long they want to use the programme for. (XA25)* |
|  | *The design considers the end user* | *That was one thing that I think was good about the programme as well was that, they were very clear, you know, with the videos and the instructions, and little symbols or things that would pop up on the screen, it was really clear what she needed to do. She never once asked me. And I never really had to instruct what was coming up on the screen, or change the way she was doing things. I thought that was good. (WT75)* |
|  | *Health professionals/exercise specialists can tailor the programme to suit their patient* | *First impressions were exceptional. As I mentioned before, the progressive nature of the exercise, being responsive to how the patient felt they were performing tasks, giving the clinicians the ability to off in the background progression regress, activities was a very exciting prospect ... (XA25)* |
| ***Study staff level facilitators of acceptability*** | | |
|  | *It is user friendly and benefits the patient* | *As a clinician, they might have a thousand things to now choose from, and they have to work out which ones. Most people will go back to something that they feel is comfortable which is a reasonably good product, and that they’ve probably had feedback from clients or patients that it’s a user friendly valuable thing. I think they’re strong incentives for clinicians. If someone gives them good feedback about a particular device or they might see someone do the program using this Standing Tall, and then they came back and they saw how much they benefited, those sort of things are also really informative, which people would be more likely to promote the product and support it and embrace it. (MP87)* |
| The programme is enjoyable and offers more than just physical activity to the user | *Having a solid evidence base drives interest in StandingTall* | *After the publication of article in BMJ the team got a flurry of enquiries from physiotherapists wanting to use the tool (ET76).* |
|  | *What does the patient get out of using StandingTall* | *We always forget about talking about it from the perspective of the patient. Probably really focusing on that and sort of feeding that back into the system of what people get out of it, assuming a lot of it’s positive. So that that can then support it more as an option in the system (UD15).* |

## **Supplementary Table 7.** What were the barriers and facilitators to telehealth delivery (because of COVID-19)

Represented at the participant, health professional/exercise specialist, health service manager and study staff levels

| **Barriers to telehealth** | | |
| --- | --- | --- |
| ***Participant level barriers to telehealth delivery*** | | |
| Technology presents a barrier to using telehealth | *StandingTall* is not compatible with android devices | *Oh, I’m pretty tech savvy, not very happy with the App, it’s not compatible with Android devices. So, I cannot use it on either my mobile phone or my tablet, I’m stuck with my laptop, so, that’s a pretty major issue I think. (5481GYL)* |
| Support is critical to overcoming barriers | *Without support, the set-up would be too hard* | *It was a bit hard really because I had to do a Zoom call with [the health professional/exercise specialist] and it took ages to get me actually onto the programme and you got onto the homepage which said the exercise programme and you went onto how much exercise you thought you could do. (HT004)* |
|  | *Not knowing why you are doing some exercises; there is no way to check* | *I think it's essential to have somebody look at the way you're doing things because everyone will interpret things differently. And this is what I thought, I didn't have anyone to look at how I was doing things as to whether like, you know with like a physio background I think I was doing things okay, but you don't know with your own body. And I wasn’t anywhere where there was a mirror, so I couldn’t look. (5134APM)* |
| ***Health professional/exercise specialist level barriers to telehealth delivery*** | | |
| Technology presents a barrier to using telehealth | *Having the right technology* | *Initially we had issues with access to Wi-Fi, even though there was a hospital Wi-Fi, at the time, we didn’t have functionality for guests to join the Wi-Fi, so we had to get a separate modem and get access to the Wi-Fi for setting up the patients that way. So, that was a technological challenge. (FJ23)* |
|  | *Does it raise issues of equity?* | *I'm putting this down to a couple of different factors, one of which is poor connectivity throughout the [region], we still have many families without the internet, myself being one of them, you know, I can't get a good signal at my own home, so we have people on, and we have the lowest socio-economic demographic of the whole [district]. (XA25)* |
|  | *It relies on stable internet* | *That was the other thing too, you know … I don’t know if it was the internet or whatever. You know when they would do the demo and either have it work or demo, I mean like it was just…it took ages, buffered and buffered and buffered. (GG3)* |
| The users technical literacy influences confidence and ease of use | *Technical literacy of the user is a barrier, and they need to be supported* | *So, I think for most of those who haven’t done it, who haven’t done it at all, was the IT problem, getting it to work or to make it work, not because it wasn’t working, but because of their limited ability to deal with IT and computers and tablet and stuff like that, that was one of the big problems. (GG2)* |
|  | *The first session (set-up) is daunting and too much for some people* | *The other person that we did get on that was really interested, I never got her to start the programme because I think the paperwork was too intensive at the beginning and it scared her, it put her off. So, obviously, like, [] just, she could do it, and because we couldn’t sit with them to help them out, we had to do everything online over Zoom, and it was challenging in that sense. (FF1)* |
| It requires changing current practice, which is hard | *It was scary to move falls patients online* | *I guess at first we thought we were going to do it in like a clinic based thing together. And then it turned into being online, which both of us had our concerns about because obviously, if you're working at an at risk falls population, doing exercise over a screen while you're not there to physically observe and support, at first it was a little bit yeah, I guess, something we weren't used to. (HV49)* |
|  | *It has a big impact on existing workload* | *Yeah, time consuming, and it just requires me to problem solve and do IT stuff which I just didn’t view as my core business. (VU92)* |
|  | *Clinicians are time poor, which made setting up the client challenging* | *Right, well, the concept was good. We liked the idea of it. The set up was quite hard and long-winded and a lot of the exercises are repeated a lot, which I found was doing the set-up, would take quite a while with the patients. So, keeping the patients motivated during the set-up as well was quite hard ... (KK1)* |
|  | *I lacked confidence as the facilitator of StandingTall* | *I think, for me, it would be, and maybe this is the reflection on me, but having a clear pathway of the stages that we need to do, you know, so the initial form-filling and then the setting up of IT and going back into some of the actual software and just, for me, to be confident in that as a facilitator, really. (TT1)* |
| Support is critical to overcoming barriers | *Patients need to be supported to stay on track* | *I found that you’ve…I was asking people, you know, from the follow-up call and they hadn’t logged on yet. Oh, I’m going to do it, sort of thing (KK1)* |
| ***Health service manager level barriers to telehealth delivery*** | | |
| Technology presents a barrier to using telehealth | *Access to Wi-Fi* | *One of the things that would be a barrier, and sort of has been a bit, is the Wi-Fi in the hospitals. The hospitals have a very low firewall threshold. (LK38)* |
|  | *Equity and the digital divide* | *That suits some people and you’re really narrowing down and you're probably creating quite an equity issue in terms of the people that suit that are probably your more educated, more well off people at the first place. (EY63)* |
| The users technical literacy influences confidence and ease of use | *IT skills of the user* | *Particularly if the person isn't tech-savvy? I mean, that's, that's the issue we've had with all of our programmes, you know, this age group. Access is an issue for a start with StandingTall, some people simply can’t, they don't have the equipment, or internet access. Even if do, there’s that barrier around the literacy of being able to use all of the systems involved. (EY63)* |
| Support is critical to overcoming barriers | *There is a need for more support at home* | *And then overlay, overlaying COVID on top of that too, where we would have hoped that we could have recruited family members to set, set things or be there when they do the programme online, you know, be a son or a daughter next to them to get them using the smartphone or the iPad. That has not, we've not been able to do that in our extended lockdown, lockdowns. So that's really impacted the rollout. (AC12)* |
| ***Study staff level barriers to telehealth delivery*** | | |
| Technology presents a barrier to using telehealth | *Having the right technology* | *The technology. The technology can be difficult. We’ve had issues with things like – it’s funny because a lot of these things in the Covid world has changed now. One thing I think we’ve talked about before, in one of hospitals where I started doing set-ups for the physio, there was guest wi-fi which was fine. So then in the other hospital when I went to do it there, there was no guest wi-fi. So without guest wi-fi you can’t do a set-up in hospital, not possible. So that was an interesting little challenge or barrier (UD15)* |
|  | *Does it raise issues of equity?* | *We had this idea here that no one would participate because they don’t have iPads and they can’t afford iPads and kind of having to steer them away from it, it doesn’t need to be an iPad. They can use a desktop computer, or they can use an android galaxy, something cheaper than iPad. There is those complications of them trying to work out how to set someone with a device too and access a device. (ZG40)* |
| The users technical literacy influences confidence and ease of use | *IT skills of the user* | *You’re given an App and you’ve got to do this. For some people, that’s all a piece of cake, they just take it all up and they’re fine and they power away and there’s no problem. But for other people it can be really challenging, like some parts of it can be really challenging with the technology or the understanding of how to do the training and other things (UD15)* |
| **Facilitators of telehealth** | | |
| ***Participant level facilitators of telehealth delivery*** | | |
| Technology allows for people to maintain their health (behaviours) | *Grateful to have a programme they could access during COVID-19 lockdowns* | *I could manage it pretty easily and, but I wasn’t actually bored with it, I just did it because you know, it was there to do. And I thought well, it’s helping me in some ways, so I just did it. I can't say I actually felt bored or that it was a big issue at all. You know, in many ways I was just grateful to have something to do. (5457KNH)* |
|  | *StandingTall was a source of entertainment and people were looking for something to do* | *And sometimes in lockdown, I knew I could, I knew I would be training. Just to keep myself entertained. (5048XHB)* |
| Having support lowers the barrier to using technology | *The implementation officers support and phone calls help keep us engaged* | *She’s just rung and told me how well I'm doing and everything. It’s been motivating as well, because I sometimes think, ‘oh no, I’m not doing that as well.’ And it just gives you a lift to carry on and keep on at it. (5048XHB)* |
| ***Health professional/exercise specialist level facilitators of telehealth delivery*** | | |
| COVID-19 lowered the barrier to using technology | *COVID-19 may have driven take up of the programme* | *StandingTall has been great for them but some of them are really afraid they won’t qualify for StandingTall. But, yes, if it wasn’t…if COVID didn’t happen, I think it would have been probably harder to…you know (GG3)* |
| Having support lowers the barrier to using technology | *Family support lowers the barrier to using technology* | *I mean, we do have low health literacy. We have generally a older population, more 70 to 80 years than 60. We do have limitations in their use of technology. We do have people who are trying to do telehealth we saw along through COVID. We can only do that via a telephone call. They are not able to access emails or video links. Even if they've actually got a device that accepts a video link, they can't work it. So there would be some barriers there. But that’s for a proportion of people. And then obviously, there'd be a proportion that could do it or that they have family members that would help them to do it. I think once the thing’s loaded on and someone’s shown them how to work it, it's a bit easier (GH61).* |
| ***Health service manager level facilitators of telehealth delivery*** | | |
| Technology allows for people to maintain their health (behaviours) | *Keen to utilise technology to improve health* | *generally our health district is very keen to utilise technology. Our region is geographically dispersed and does contain quite rural places and we one of the fastest largest growing ageing population in Australia, particularly our Tweed Local Government Area. So, the fact that our population demographics and geography and our goals around better utilising around technology to provide care to people and keep them out of hospital really fit neatly with the Standing Tall programme. (KP27)* |
| COVID-19 lowered the barrier to using technology | *COVID-19 removed the barriers to eHealth* | *I think Covid’s probably helped now in terms of getting physios and OTs to see this as part of their core business because it can be done online and in people’s homes. That’s kind of been a positive (RD52)* |
| ***Study staff level facilitators of telehealth delivery*** | | |
| COVID-19 lowered the barrier to using technology | *COVID-19 removed the barriers to eHealth* | *Yeah. I think Covid has been a real game changer because clinicians have had to consider different models of care and different ways of getting services to people, so they’ve been forced to actually consider Telehealth options, internet options, remote YouTube exercises, all those sorts of things. They’ve had to actually embrace it. (MP87)* |
| Having support lowers the barrier to using technology | *Family support lowers the barrier to using technology* | *She’s totally willing and physically like totally capable too. But just the technology has been too much for her. There is people like that, and she doesn’t have a support person. If she had someone to help her with it it’d be fine, because I’ve got a 92 year old man that’s probably not very tech savvy either, but he’s got a very strong support system in his family and he’s been fine. (UD15)* |
|  | *Access to a help centre* | *I think this telehealth stuff is going to be probably a bit of an enabler, if it’s done properly. ... I think the consideration, in the longer term, how this works as in how the support is provided both not only exercise specialist support or support around the programme but also the technological stuff, like where do people go to get a glitch fixed, because they can’t get into their App. If you take that out of the equation, where does that happen, and how does that happen and what’s the need for it to happen and is there different needs for different people. Like I said before, if there’s somewhere they need to have very close contact with a physio to monitor and is there somewhere it could just be almost like a get healthy service thing, where you’ve got a 1300 number and you’ve got a coach on the end. You can just ring up and say I’m having problem with this exercise or this technological problem and it gets fixed. But there needs to be some kind of ongoing support for people that are using it, ... (UD15)* |

## Supplementary Table 8. Survey outcomes for 3- and 6-months

| Outcome, n(%) | 3-month survey  n=172^a^ | 6-month survey  n=158^b^ |
| --- | --- | --- |
| Change in overall balance |  |  |
| No change (or condition has gotten worse) | 13 (8) | 21 (13) |
| Almost the same, hardly any change at all | 31 (18) | 17 (11) |
| A little better, but no noticeable change | 36 (21) | 17 (11) |
| Somewhat better, but the change has not made any real difference | 18 (11) | 14 (9) |
| Moderately better, and a slight but noticeable change | 40 (23) | 50 (32) |
| Better and a definite improvement that has made a real and worthwhile difference | 28 (16) | 35 (22) |
| A great deal better and a considerable improvement that has made all the difference | 6 (4) | 4 (3) |
| Overall, I would rate StandingTall as:^c^ |  |  |
| Poor | 5 (3) | 2 (1) |
| Fair | 29 (17) | 30 (19) |
| Good | 129 (75) | 68 (43) |
| Excellent | 8 (5) | 57 (36) |
| I find StandingTall is easy to use |  |  |
| Disagree | 6 (4) | 8 (5) |
| Tend to disagree | 9 (5) | 5 (3) |
| Tend to agree | 52 (30) | 43 (27) |
| Agree | 105 (61) | 102 (65) |
| I like to use the StandingTall programme |  |  |
| Disagree | 9 (5) | 13 (8) |
| Tend to disagree | 19 (11) | 25 (16) |
| Tend to agree | 61 (36) | 48 (30) |
| Agree | 83 (48) | 72 (46) |
| I feel confident about doing the StandingTall exercises |  |  |
| Disagree | 4 (2) | 6 (4) |
| Tend to disagree | 6 (4) | 3 (2) |
| Tend to agree | 36 (21) | 29 (18) |
| Agree | 126 (73) | 120 (76) |
| I learnt to use StandingTall in a short space of time |  |  |
| Disagree | 4 (2) | 5 (3) |
| Tend to disagree | 6 (4) | 3 (2) |
| Tend to agree | 50 (29) | 42 (27) |
| Agree | 112 (65) | 108 (68) |
| I still need some technical help to use StandingTall program |  |  |
| Disagree | 100 (59) | 94 (60) |
| Tend to disagree | 32 (19) | 29 (18) |
| Tend to agree | 24 (14) | 19 (12) |
| Agree | 15 (9) | 16 (10) |
| The instructions are easy to follow |  |  |
| Disagree | 3 (2) | 4 (3) |
| Tend to disagree | 16 (9) | 8 (5) |
| Tend to agree | 57 (33) | 54 (34) |
| Agree | 96 (56) | 92 (58) |
| The instructions are helpful |  |  |
| Disagree | 5 (3) |  |
| Tend to disagree | 9 (5) |  |
| Tend to agree | 52 (30) |  |
| Agree | 106 (62) |  |
| The instructions for using the StandingTall program are |  |  |
| Much too complex | 5 (3) | 5 (3) |
| A little too complex | 10 (6) | 6 (4) |
| About right | 127 (74) | 120 (76) |
| A little too basic | 21 (12) | 17 (11) |
| Much too basic | 2 (1) | 4 (3) |
| N/A – not used them | 7 (4) | 5 (3) |
| Have you ever felt like you needed help to use the StandingTall program in the last 3 months |  |  |
| No | 121 (71) | 125 (79) |
| Yes | 50 (29) | 33 (21) |
| Devices used for StandingTall (could select more than one) |  |  |
| iPad | 79 (46) | 66 (42) |
| Laptop | 44 (26) | 42 (17) |
| Desktop computer | 33 (19) | 30 (19) |
| Smart TV | 2 (1) | 2 (1) |
| Android tablet | 21 (12) | 15 (10) |
| For you, has using a computer, tablet or other electronic device for the StandingTall exercises been.... |  |  |
| Very difficult | 4 (2) | 4 (3) |
| Somewhat difficult | 9 (5) | 9 (6) |
| A little difficult | 30 (18) | 19 (20) |
| Not difficult | 126 (74) | 125 (79) |
| Unsure | 1 (1) | 1 (1) |
| Now that you have tried the StandingTall program, will you continue using your electronic device for the StandingTall exercises? |  |  |
| Yes, definitely | 120 (71) | 89 (56) |
| Yes, but still a little uncomfortable using the device | 11 (7) | 7 (4) |
| No, I quite like doing the exercises but would prefer to do in a different way without using technology | 12 (7) | 22 (14) |
| No, I will not continue | 25 (15) | 40 (25) |
| Did you have any difficulties: |  |  |
| 1. Getting access to a device to use StandingTall on | 17 (10) | n/a |
| 1. Getting registered to use StandingTall | 13 (18) | n/a |
| 1. Doing your first exercise session/s | 30 (18) | n/a |
| 1. Doing your exercise sessions | n/a | 32 (20) |
| 1. Doing 2 hours of exercise a week using StandingTall | 82 (48) | 78 (50) |
| 1. Getting the help you need when you need it | 32 (19) | 21 (14) |
| In future, would you be willing to pay a small one-off amount of $5/£3 to access the StandingTall program? |  |  |
| No | 46 (27) | 49 (31) |
| Yes | 84 (49) | 72 (46) |
| Maybe | 42 (24) | 37 (23) |
| In future, would you be interested in accessing the program and advice from a helpline service as part of an annual subscription? |  |  |
| No | 75 (44) | 75 (48) |
| Yes | 15 (9) | 26 (17) |
| Maybe | 81 (47) | 56 (36) |

^a^149/184 (81%) from Australia and 23/62 (37%) from Northern England

^b^142/184 (77%) from Australia and 16/62 (26%) from Northern England

^c^n=157
